# BDH1 Mediates Aerobic Exercise-Induced Improvement in Skeletal Muscle Metabolic Remodeling in Type 2 Diabetes Mellitus

**DOI:** 10.3390/biom16010115

**Published:** 2026-01-08

**Authors:** Mingyu Wu, Xiaotong Ma, Wei Dai, Ke Li, Haoyang Gao, Yifan Guo, Weihua Xiao

**Affiliations:** 1 Shanghai Key Lab of Human Performance, School of Sports and Health, Shanghai University of Sport, Shanghai 200438, China; 2321517005@sus.edu.cn (M.W.); 2321518007@sus.edu.cn (X.M.); 2321518018@sus.edu.cn (W.D.); 2411517002@sus.edu.cn (K.L.); 2411516001@sus.edu.cn (H.G.); 2 School of Elderly Care Services and Management, Nanjing University of Chinese Medicine, Nanjing 210023, China; yifan_guo@njucm.edu.cn

**Keywords:** type 2 diabetes mellitus, skeletal muscle, exercise, metabolic remodeling, mitochondria, glycolipid metabolism, inflammation, oxidative stress, fibrosis

## Abstract

Background: Type 2 diabetes mellitus (T2DM) is typically characterized by the dysregulation of metabolic remodeling. As a systemic metabolic disease, T2DM can affect the mass and function of skeletal muscle by inducing impaired energy metabolism, mitochondrial dysfunction, and chronic low-grade inflammation. β-Hydroxybutyrate dehydrogenase 1 (BDH1) is a rate-limiting enzyme involved in ketone body metabolism, and its activity is down-regulated in various models of diabetic complications. Aerobic exercise (AE) is recognized as an effective intervention to promote energy homeostasis and alleviate metabolic stress. Whether its protective effect on skeletal muscle in T2DM involves the regulatory control of BDH1 expression remains unclear. Methods: Wild-type (WT) and systemic BDH1 knockout (BDH1^−/−^) male C57BL/6J mice were used to establish the sedentary control (SED) and AE models of T2DM by providing a high-fat diet combined with streptozotocin injection. The indicators related to metabolic remodeling were detected by hematoxylin and eosin staining, immunofluorescence staining, quantitative real-time PCR, and Western blot assays. Results: After 8 weeks of AE, we found that AE improved glycolipid metabolic disorders and mitochondrial quality control in the gastrocnemius muscle of T2DM mice by up-regulating BDH1, thereby alleviating oxidative stress, inflammation, and fibrosis. Compared with the WT mice, the BDH1^−/−^ T2DM mice in the SED group exhibited more severe phenotypic impairment. The metabolic improvement effect of AE was attenuated in the BDH1^−/−^ mice. Conclusions: BDH1 is a key effector enzyme that may mediate the AE-induced improvement in metabolic remodeling in the gastrocnemius muscle of mice with T2DM.

## 1. Introduction

Type 2 diabetes mellitus (T2DM), a metabolic disease characterized by chronic hyperglycemia, impairs the health and well-being of approximately 14% of the global population [1]. Insulin resistance and β-cell dysfunction are key factors mediating T2DM development, and its in-depth pathological mechanisms include β-cell dedifferentiation, mitochondrial dysfunction, and oxidative stress [2]. Chronic hyperglycemia, together with multiple pathological effects such as pancreatic β-cell dysfunction, immune inflammation, and fibrosis, collectively promotes T2DM progression [3]. Abnormal lipid droplet accumulation mediated by T2DM is a key mediator triggering ectopic lipid deposition and lipotoxicity, which exacerbate T2DM and its complications in major target organs including skeletal muscle, heart, and kidney [4].

Metabolic remodeling involves multidimensional alterations in vital biological functions, such as glycolipid metabolism, and mitochondrial quality while integrating regulatory trends in metabolic enzyme activity, transcription factors, and signaling pathways. T2DM progression is closely associated with nutritional excess and energy imbalance. As a vital locomotor organ that responds to energy expenditure and nutrient supply, skeletal muscles trigger corresponding metabolic alterations involving insulin resistance, lipid deposition, mitochondrial functional imbalance, and transcriptional remodeling of key metabolic pathways [5,6]. They also regulate systemic blood glucose and serve as a key target tissue for glucose disposal in response to insulin stimulation [7]. Skeletal muscle insulin resistance represents the core pathological mechanism underlying impaired metabolic remodeling: It hinders energy utilization and exacerbates metabolic syndrome [5]. Furthermore, the impaired ability of skeletal muscle to regulate glycolipid metabolism induces an imbalance in mitochondrial fusion and fission. This imbalance reduces the cell’s adaptability to substrate oxidation, thereby promoting lipotoxic accumulation in muscle cells and forming a vicious cycle of insulin resistance [8]. Moreover, the accumulation of diacylglycerol in skeletal muscles induced by nutrient excess and sedentary behavior triggers inflammation, oxidative stress, and fibrosis, thereby impairing the efficiency of skeletal muscle in responding to metabolic demands [9]. Lifestyle interventions, including low-energy diets and moderate-intensity aerobic exercise (AE), confer significant benefits for blood glucose control and weight loss programs in patients with T2DM [10]. Among these interventions, physical exercise has been demonstrated to increase glucose uptake in skeletal muscle through the mediating effect of glucose transporter 4 (Glut4); it can also reduce lipid products in skeletal muscle and enhance lipid oxidative capacity [11]. Mitochondrial biogenesis and mitochondrial dynamics regulate the quality of the mitochondrial network in muscles. Mitochondrial processes in skeletal muscles are collectively mediated by factors such as peroxisome proliferator-activated receptor gamma coactivator 1 alpha (PGC-1α), peroxisome proliferator-activated receptors, nuclear respiratory factors, and transcription factor A, mitochondrial (TFAM). As a physiological stimulus that promotes muscle energy expenditure, exercise can significantly improve mitochondrial phenotypes in muscles [12,13,14]. Inflammatory cytokines, stress kinases, and reactive oxygen species (ROS) disrupt insulin signaling, leading to glucose metabolism disorders and organ dysfunction [15]. Among these pathological changes, inflammatory infiltration in skeletal muscles often leads to fibrosis, muscle atrophy, and impaired myocyte function. AE has been proven to help resist inflammation and fibrosis in skeletal muscles [16].

**Figure 1:**
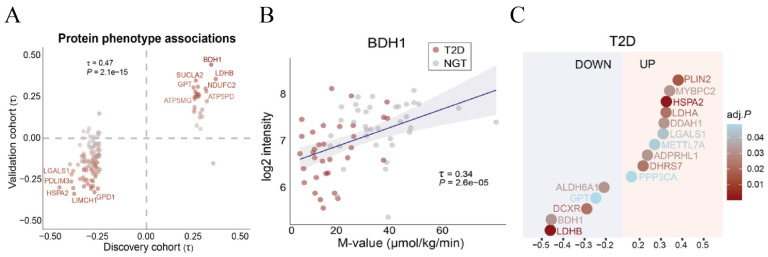
Proteomic signature of insulin-sensitive and -resistant skeletal muscle. (**A**). Correlation analysis of protein phenotype associations in the discovery cohort and validation cohort. (**B**). Kendall’s rank correlation analysis between baseline BDH1 protein abundance and individual insulin sensitivity (M-value). (**C**). Differential protein expression in the discrete comparison between patients with T2DM and individuals with normal glucose tolerance. Reproduced from [18].

β-Hydroxybutyrate dehydrogenase 1 (BDH1), a member of the BDH protein family, is a short-chain dehydrogenase localized to the inner mitochondrial membrane and plays a key rate-limiting role in ketone body synthesis [17]. Correlation analysis data from protein phenotype association studies in a discovery cohort and a validation cohort—comprising patients with T2DM and individuals with normal glucose tolerance—showed that BDH1 is up-regulated in biopsied muscle tissues from individuals with high insulin sensitivity (Figure 1A) [18]. Scatter plots depicting BDH1 protein expression under different insulin sensitivity (M-value) conditions and the differential protein expression between patients with T2DM and individuals with normal glucose tolerance revealed that BDH1 is strongly positively correlated with insulin sensitivity (M-value) and is significantly down-regulated in the skeletal muscles of patients with T2DM (Figure 1B,C) [18]. Mendelian randomization analysis mediated by plasma proteomics revealed that BDH1 is a promising target for T2DM treatment [19]. BDH1 overexpression exerts protective effects to alleviate inflammation, oxidative stress, fibrosis, and glucose metabolism disorders, whereas BDH1 deficiency impairs skeletal muscles’ exercise endurance [17,20]. In hepatic mitochondria, BDH1 catalyzes the final step of β-hydroxybutyrate (β-HB) synthesis; in extrahepatic tissues, it acts as a key enzyme facilitating β-HB degradation. BDH1 maintains β-HB homeostasis during fasting and exercise; its knockout impairs exercise-induced endogenous nutritional ketosis and attenuates the regulatory role of BDH1-dependent β-HB production as a signaling molecule in improving cerebral cognitive function [21]. Exercise has been proven to enhance the oxidation rate of ketone bodies in skeletal muscles. The β-HB oxidation rate in the muscle of exercised rats is approximately twice that of sedentary controls, accompanied with increased BDH1 activity in the gastrocnemius muscle [22]. β-HB, a byproduct of lipid metabolism, serves as an alternative energy source during exercise [23]. In one study, C2C12 myocytes were exposed in vitro to forskolin—a cAMP pathway agonist that mimics exercise effects—and the β-HB levels in cell lysates were found to be higher than those in the control cells [24]. In addition, β-HB is an exercise mimetic; it further inhibits the proinflammatory M1 phenotype of macrophages, thereby ameliorating lipid deposition and PANapoptosis in senescent hepatocytes [23]. Sixteen weeks of treadmill AE up-regulated circulating β-HB levels and induced the expression of BDH1, hydroxycarboxylic acid receptor 2 (HCAR2), and PPARG in skeletal muscle, effectively delaying aging-related sarcopenia [13]. Another study also revealed that AE, by regulating the β-HB/HCAR2/PPARγ signaling pathway, also up-regulates the expression of brain-derived neurotrophic factor and alleviates neuroinflammation—this process also involves the up-regulation of BDH1, a protein associated with ketone body synthesis [21]. However, whether BDH1 plays a key role in AE-induced improvements in skeletal muscle adaptation and metabolic remodeling in T2DM mice remains to be verified. By establishing T2DM models in wild-type (WT) mice and mice with systemic BDH1 knockout (BDH1^−/−^), we investigated the core role of BDH1 in the 8-week AE-induced alleviation of skeletal muscle glycolipid metabolism disorders, mitochondrial homeostasis imbalance, oxidative stress, inflammation, and fibrosis in T2DM mice.

## 2. Materials and Methods

### 2.1. Animal Experiments

In this study, 4-week-old C57BL/6J male mice were used as research subjects. WT C57BL/6J mice were purchased from the Institute of Animal Models, Nanjing University. Systemic BDH1 knockout (BDH1^−/−^) mice were generated on the C57BL/6JGpt genetic background using CRISPR/Cas9 gene-editing technology (GemPharmatech, Nanjing, China). The knockout strategy in this study targeted the mouse Bdh1-203 transcript (ENSMUST00000115227.9), which represents the longest and most representative protein-coding isoform of the BDH1 gene. Two specific single-guide RNAs (sgRNAs) were designed to target and delete the genomic region spanning exon 3–6 of the BDH1 gene. This region covers a coding sequence of approximately 479 bp, which contains the conserved catalytic domain of the BDH1 protein. The sgRNA sequences used were as follows: gRNA1 (5′-CAGTGCATAGCCGGCCGATC-3′, PAM: TGG) and gRNA2 (5′-TAGGAAGGAACATGACGTCA-3′, PAM: TGG). After in vitro transcription, the aforementioned sgRNAs were comicroinjected with Cas9 protein into fertilized zygotes of C57BL/6JGpt mice, which were then transplanted into pseudopregnant female mice to generate F0-generation mice. Positive F0 mice were identified via genotyping and subjected to successive breeding. Ultimately, BDH1^−/−^ mice with a stable genetic background were obtained and used for subsequent experimental studies (provided by Jiangsu Jicui Yaokang Biotechnology Co., Ltd., Nanjing, China). All mice were housed in the Laboratory Animal Center of Shanghai University of Sport under controlled conditions (temperature maintained at 21–25 °C, humidity at 40–50%, and a 12 h light/12 h dark cycle). Food intake of the mice was recorded daily, and intervention was initiated after 1 week of adaptive feeding. All experimental protocols in this study were approved by the Scientific Research Ethics Committee of Shanghai University of Sport (Approval No. 102772019DW009).

In reference to our previously published articles [25,26], a T2DM mouse model was established by feeding mice a 12-week high-fat diet (HFD; D12492, 60% fat-derived energy, SYSE Ltd., Nanjing, China) combined with a single intraperitoneal injection of streptozotocin (STZ) at a dose of 100 mg/kg (S0130, Sigma–Aldrich, St. Louis, MO, USA). Mice with a fasting blood glucose level ≥ 16.7 mmol/L were considered successfully modeled as T2DM mice. The successfully modeled T2DM mice were divided into four groups: diabetic sedentary group (WT-SED), diabetic aerobic exercise group (WT-AE), BDH1 knockout diabetic sedentary group (BDH1^−/−^-SED), and BDH1 knockout diabetic aerobic exercise group (BDH1^−/−^-AE). In addition, the normal diet WT C57BL/6J mice of the same age fed with normal diet were used as a healthy control group (WT-CON).

### 2.2. Aerobic Exercise Protocol

The mice in the AE group first underwent a 5-day adaptive training phase, which was performed at a speed of 12 m/min for 15–20 min per day (0° incline). After the adaptation period, the mice were given 2 days of rest. Subsequently, they performed treadmill exercise (0° incline) at a speed of 15 m/min for 60 min per session, 5 days a week (Monday to Friday) for 8 weeks.

### 2.3. Tissue Collection

After the 8-week AE, the mice’s body composition, glucose tolerance test, and insulin tolerance test were measured [27]. Following these measurements, the mice were anesthetized with 2% isoflurane and then euthanized by cervical dislocation. Bilateral gastrocnemius muscles were quickly excised on an ice box using autoclaved scissors and forceps. A portion of the gastrocnemius muscle was placed in muscle fixation solution for morphological analysis, and the remaining part was rapidly frozen in liquid nitrogen and stored at −80 °C.

### 2.4. Hematoxylin and Eosin (H&E) Staining

Gastrocnemius muscle samples were removed after being immersed in muscle fixation solution for 48 h. After being washed with phosphate-buffered saline (PBS) and pure water, the samples were subjected to gradient ethanol dehydration, xylene clearing, and paraffin infiltration to prepare paraffin blocks. These blocks were then cut into 5 μm thick paraffin sections using a microtome. The sections were dewaxed with xylene and rehydrated through a gradient ethanol series, followed by staining in H&E jars. After subsequent dehydration, clearing, and mounting with neutral balsam, the sections were observed under an OLYMPUS microscope, and images were captured from different visual fields.

### 2.5. Immunofluorescence Staining

The paraffin sections were dewaxed, rehydrated, and washed with pure water. The pure water was then replaced with antigen retrieval solution, and the sections were heated in a microwave oven. After heating, the sections were allowed to cool naturally to room temperature and then blocked with immunofluorescence staining blocking solution. Following blocking, the sections were incubated overnight at 4 °C with primary antibodies against α-smooth muscle actin (α-SMA; 1:300, Santa Cruz Biotechnology, Dallas, TX, USA, sc-53142) and mitochondrial fission 1 protein (Fis1; 1:300, Proteintech, Wuhan, China, 10956-1-AP). On the next day, the sections were washed with PBS and then incubated in the dark with the corresponding secondary antibodies: Alexa Fluor^®^ 488-conjugated goat anti-rabbit IgG (H + L) (Cat. No. ZF-0511, Zhongshan Jinqiao, Beijing, China) and Donkey Anti-Mouse IgG H&L (Alexa Fluor^®^ 555) (Cat. No. ab150106, Abcam, Cambridge, UK). Afterward, the sections were mounted using anti-fluorescence quenching mounting medium containing 4′,6-diamidino-2-phenylindole. Finally, the sections were observed under a confocal microscope, and images were captured and stored from different visual fields.

### 2.6. Quantitative Real-Time PCR (qRT-PCR)

Tissue samples stored in a −80 °C refrigerator were retrieved, and approximately 30 mg of gastrocnemius muscle was weighed. Total RNA was extracted from the skeletal muscle using the Trizol method. In accordance with the instructions of the cDNA Synthesis Kit (AG11706, ACCURATE BIOTECHNOLOGY (HUNAN) Co., Ltd., Changsha, China), 500 ng of total RNA was used to synthesize cDNA. Quantitative real-time qRT-PCR analysis was performed using the SYBR Green Premix Pro Taq HS qPCR Kit (Rox Plus) (AG11701, ACCURATE BIOTECHNOLOGY (HUNAN) Co., Ltd., Changsha, China). The primer sequences used in this study are listed in Table 1. β-actin was selected as the reference gene because its expression level is stable across different samples. Each experimental group included *n* = 6 biological replicates. The relative expression levels were calculated using the ΔΔCt method: First, the Ct values of the target gene and β-actin were calculated to obtain ΔCt = Ct (target gene) − Ct (β-actin); then, the control group was used as the reference to calculate ΔΔCt = ΔCt (experimental group) − ΔCt (control group mean); and finally, the fold change in relative expression was determined by calculating 2^−ΔΔCt^.

### 2.7. Western Blotting

Gastrocnemius muscle samples were placed in a lysis buffer mixture (RIPA lysis buffer/PMSF/protease inhibitor/phosphatase inhibitor = 100:1:2:2), homogenized using a high-throughput homogenizer, further lysed with an ultrasonic device, and then placed on a shaker at 4 °C to ensure complete tissue lysis. After centrifugation, the protein supernatant was collected for BCA assay. On the basis of the measured protein concentration, the original protein solution was mixed with PBS and 5× loading buffer to standardize the protein concentration across all samples. The samples were heated in a metal bath at 95 °C for 10 min and then stored at −80 °C. Approximately 40 μg of protein per sample was separated by SDS-PAGE and then transferred onto a PVDF membrane. The PVDF membrane was blocked with 5% nonfat milk or BSA at room temperature for 90 min, after which the membrane was cut into strips and incubated overnight with primary antibodies on a shaker at 4 °C. The primary antibodies used in this study were as follows: α-SMA (1:1000, Santa Cruz Biotechnology, Dallas, TX, USA, sc-53142), DRP1 (1:1000, Cell Signaling Technology, Boston, MA, USA, 8570S), MFN2 (1:1000, Cell Signaling Technology, Boston, MA, USA, 9482S), p-Akt (1:1000, Cell Signaling Technology, Boston, MA, USA, 4060S), OPA1 (1:1000, Cell Signaling Technology, Boston, MA, USA, 80471S), Glut4 (1:1000, Cell Signaling Technology, Boston, MA, USA, 2213S), PI3K (1:1000, Cell Signaling Technology, Boston, MA, USA, 4249S), BDH1 (1:1000, Santa Cruz Biotechnology, Dallas, TX, USA, sc-514413), Nrf1 (1:1000, Santa Cruz Biotechnology, Dallas, TX, USA, sc-515360), Nrf2 (1:1000, Santa Cruz Biotechnology, Dallas, TX, USA, sc-365949), and β-actin (1:20000, Proteintech, Wuhan, China, 66009-1-Ig). On the following day, the primary antibodies were recovered, and the membrane was washed with TBST and then incubated with the corresponding horseradish peroxidase (HRP)-conjugated goat anti-mouse IgG (H + L) (Cat. No. A0216, Beyotime Biotechnology, Shanghai, China) or HRP-conjugated goat anti-rabbit IgG (H + L) (Cat. No. A0216, Beyotime Biotechnology, Shanghai, China) for 90 min at room temperature. After being washed with TBST, the membrane was developed and imaged using an automatic chemiluminescence imaging system. The Western blot results were analyzed using ImageJ FIJI software version 1.53 (National Institutes of Health, Bethesda, MD, USA) (*n* = 3 biological replicates).

### 2.8. Statistical Analysis

The experimental results were analyzed using GraphPad Prism 9 software. All data were presented as mean ± standard deviation (mean ± SD). One-way ANOVA was used for comparisons among three groups, followed by Tukey’s test for post hoc multiple comparisons. Differences among four groups were evaluated using two-way ANOVA combined with Tukey’s multiple comparison test. *p* < 0.05 was considered statistically significant, and *p* < 0.01 was considered extremely statistically significant.

## 3. Results

### 3.1. Aerobic Exercise Improves Mitochondrial Quality Control (MQC) and Oxidative Stress in Skeletal Muscle of T2DM Mice

**Figure 2:**
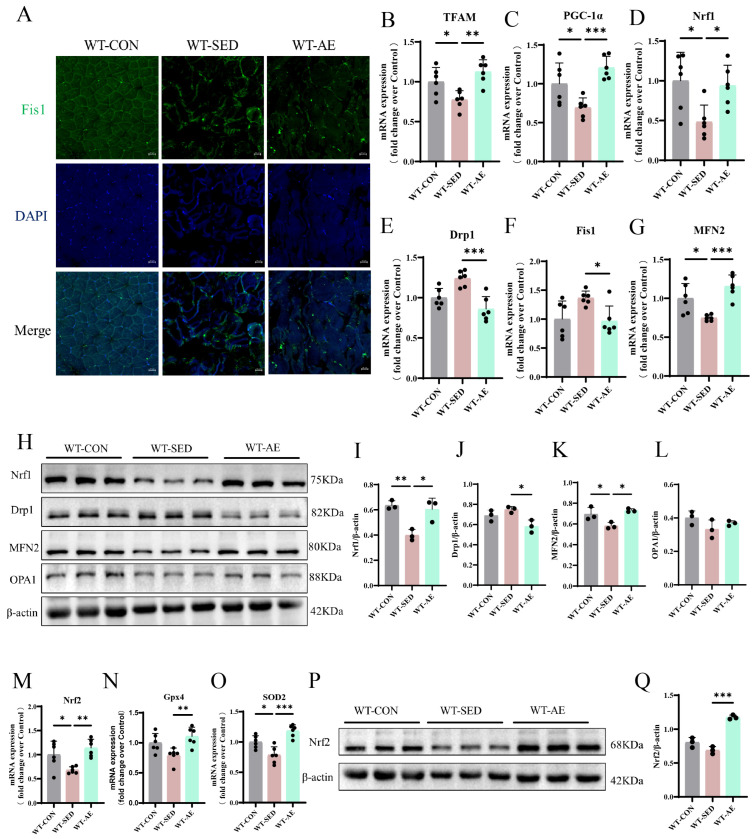
Effects of AE on MQC and oxidative stress in the gastrocnemius muscle of WT T2DM mice. (**A**). Representative images of Fis1 immunofluorescence staining in the gastrocnemius muscle. Scale bars = 20 μm. (**B**–**D**). mRNA expression levels of genes regulating mitochondrial biogenesis (TFAM, PGC-1α, Nrf1) in the gastrocnemius muscle. *n* = 6. (**E**,**F**). mRNA expression levels of genes regulating mitochondrial fission (Drp1 and Fis1) in the gastrocnemius muscle. *n* = 6. (**G**). mRNA expression level of the gene regulating mitochondrial fusion (MFN2) in the gastrocnemius muscle. *n* = 6. (**H**). Nrf1, Drp1, MFN2, OPA1, and internal control β-actin protein expressions in the gastrocnemius muscle. (**I**). Nrf1 protein level quantitative analysis. *n* = 3. (**J**). Drp1 protein level quantitative analysis. *n* = 3. (**K**). MFN2 protein level quantitative analysis. *n* = 3. (**L**). OPA1 protein level quantitative analysis. *n* = 3. (**M**–**O**). Antioxidant (Nrf2, Gpx4, and SOD2) mRNA expression levels in the gastrocnemius muscle. *n* = 6. (**P**). Nrf2 and internal control β-actin protein expressions in the gastrocnemius muscle. (**Q**). Nrf2 protein level quantitative analysis. *n* = 3. * *p* < 0.05, ** *p* < 0.01, *** *p* < 0.001. Original images of (**H**,**P**) can be found in Supplementary Materials.

Imbalanced MQC is as an early risk factor mediating the development of skeletal muscle insulin resistance in humans and rodents [28]. We detected the expression of mitochondrial biogenesis-related factors (transcription factor A, mitochondrial [TFAM], PGC-1α, and Nrf1), mitochondrial fission-related factors (dynamin-related protein 1 [Drp1] and Fis1), and mitochondrial fusion-related factors (mitofusin 2 [MFN2] and optic atrophy 1 [OPA1]) by qRT-PCR and Western blot (Figure 2B–L). The results confirmed that the gastrocnemius muscle of T2DM mice exhibited imbalanced mitochondrial homeostasis, and AE could promote mitochondrial biogenesis and fusion while inhibiting mitochondrial fission (Figure 2B–L). Immunofluorescence detection of the mitochondrial fission factor Fis1 showed a decreasing trend in the number of Fis1-positive myocytes in the skeletal muscle of T2DM mice after AE (Figure 2A). Clinical and preclinical findings involving mitochondrial dysfunction indicated that oxidative stress is a secondary phenomenon [29]. Therefore, we further detected the gene and protein expression levels of antioxidant molecules, confirming that AE can promote the expression of antioxidant molecules Nrf2, glutathione peroxidase 4 (Gpx4), and superoxide dismutase 2 (SOD2) (Figure 2M–Q). In summary, 8 weeks of AE can effectively regulate mitochondrial homeostasis and alleviate the peroxidation state in the skeletal muscles of T2DM mice.

### 3.2. Aerobic Exercise Improves Inflammation and Fibrosis in the Skeletal Muscles of T2DM Mice

**Figure 3:**
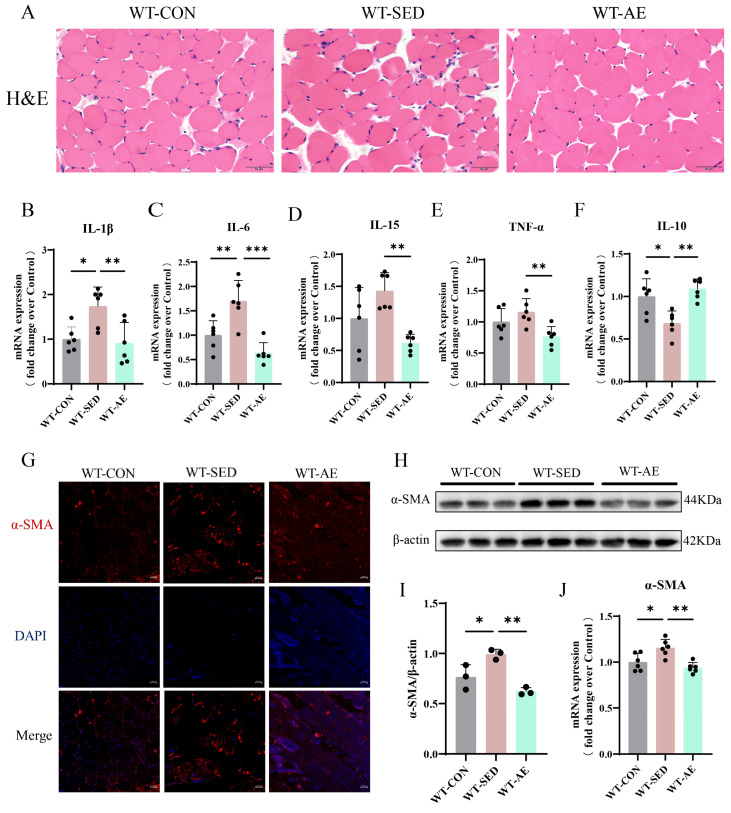
Effects of AE on inflammation and fibrosis in the gastrocnemius muscle of WT T2DM mice. (**A**). Representative images of H&E staining of the gastrocnemius muscle. Scale bars = 50 μm. (**B**–**E**). Pro-inflammatory cytokine (IL-1β, IL-6, IL-15, and TNF-α) mRNA expression levels in the gastrocnemius muscle. *n* = 6. (**F**). Anti-inflammatory cytokine IL-10 mRNA expression level in the gastrocnemius muscle. *n* = 6. (**G**). Representative images of α-SMA immunofluorescence staining in the gastrocnemius muscle. Scale bars = 20 μm. (**H**). α-SMA and internal control β-actin protein expressions in the gastrocnemius muscle. (**I**). α-SMA protein level quantitative analysis. *n* = 3. (**J**). α-SMA mRNA expression level in the gastrocnemius muscle. *n* = 6. * *p* < 0.05, ** *p* < 0.01, *** *p* < 0.001. Original images of (**H**) can be found in Supplementary Materials.

Intramyocellular lipid infiltration induced by T2DM is considered to progressively exacerbate insulin resistance by promoting the secretion of local inflammatory cytokines and the release of free fatty acids [30]. Through H&E staining, we found that the WT-SED mice showed widened myofiber gaps and mild inflammatory infiltration (Figure 3A). However, 8 weeks of AE narrowed the myofiber gaps, increased the muscle cross-sectional area, and alleviated inflammation (Figure 3A). By detecting the gene expression levels of pro-inflammatory factors interleukin-1β (IL-1β), IL-6, IL-15, and tumor necrosis factor-α (TNF-α) and anti-inflammatory factor IL-10, we found that AE improved skeletal muscle inflammatory infiltration and up-regulated the anti-inflammatory effect in WT-SED mice (Figure 3B–F). Inflammatory infiltration can induce the transdifferentiation of myoblasts into myofibroblasts, promote their excessive activation and proliferation, and ultimately trigger extracellular matrix deposition and muscle fibrosis [31]. Therefore, we further detected the positive expression area of α-SMA in the gastrocnemius muscle by immunofluorescence (Figure 3G), with additional quantification of its protein and gene expression (Figure 3H–J). The results showed that α-SMA was up-regulated in the skeletal muscles of WT-SED mice, and AE inhibited its expression. In conclusion, AE can inhibit skeletal muscle inflammatory infiltration and fibrosis and enhance the anti-inflammatory effect in T2DM mice.

### 3.3. Aerobic Exercise Up-Regulates BDH1 and Improves Glycolipid Metabolism in the Skeletal Muscles of T2DM Mice

**Figure 4:**
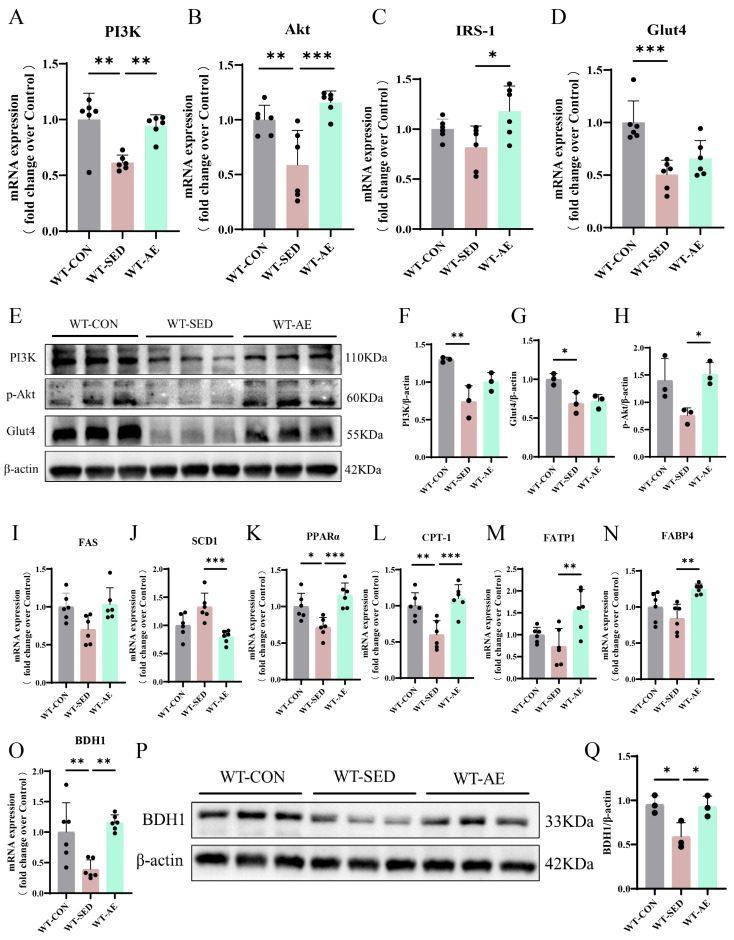
Effects of AE on glycolipid metabolism in the gastrocnemius muscle of WT T2DM mice. (**A**–**D**). Glucose metabolism-related indicator (PI3K, Akt, IRS-1, and Glut4) mRNA expression levels in the gastrocnemius muscle. *n* = 6. (**E**). PI3K, p-Akt, Glut4, and internal control β-actin protein expressions in the gastrocnemius muscle. (**F**). PI3K protein level quantitative analysis. *n* = 3. (**G**). Glut4 protein level quantitative analysis. *n* = 3. (**H**). P-Akt protein level quantitative analysis. *n* = 3. (**I**–**N**). Lipid metabolism-related indicator (FAS, SCD1, PPARα, CPT-1, FATP1, and FABP4) mRNA expression levels in the gastrocnemius muscle. *n* = 6. (**O**). BDH1 mRNA expression level in the gastrocnemius muscle. *n* = 6. (**P**). BDH1 and internal control β-actin protein expressions in the gastrocnemius muscle. (**Q**). BDH1 protein level quantitative analysis. *n* = 3. * *p* < 0.05, ** *p* < 0.01, *** *p* < 0.001. Original images of (**E**,**P**) can be found in Supplementary Materials.

Compared with the normal diet controls, the WT-SED mice exhibited glycolipid metabolism disorders. Exercise can improve skeletal muscle insulin sensitivity and affect bioactive lipid metabolism [32]. We further investigated the impact of AE on the expression of genes and proteins related to glycolipid metabolism in T2DM skeletal muscles via qRT-PCR and Western blot. The results demonstrated that AE promoted the expression of phosphatidylinositol-3-kinase (PI3K), protein kinase B (Akt), insulin receptor substrate-1 (IRS-1), and Glut4 (Figure 4A−H). It also induced the expression of lipid oxidation genes PPARα carnitine palmitoyltransferase-1 (CPT-1) and lipid transport genes fatty acid transport protein 1 (FATP1) and fatty acid binding protein 4 (FABP4) while inhibiting the expression of lipid synthesis genes fatty acid synthase (FAS), stearoyl-CoA desaturase 1 (SCD1) (Figure 4I–N).

Our previous studies indicated that AE can up-regulate BDH1 in diabetic lung tissues [17]. In the present study, we detected BDH1 expression in the skeletal muscles of T2DM mice using qRT-PCR and Western Blot (Figure 4O–Q). The results showed that AE up-regulated BDH1 expression (Figure 4O–Q). On the basis of the above findings, we speculate that 8 weeks of AE may improve skeletal muscle glycolipid metabolism disorders and MQC in T2DM mice by promoting BDH1 expression.

### 3.4. BDH1^−/−^ Attenuates the Beneficial Effects of Aerobic Exercise on MQC and Oxidative Stress in the Skeletal Muscles of T2DM Mice

**Figure 5:**
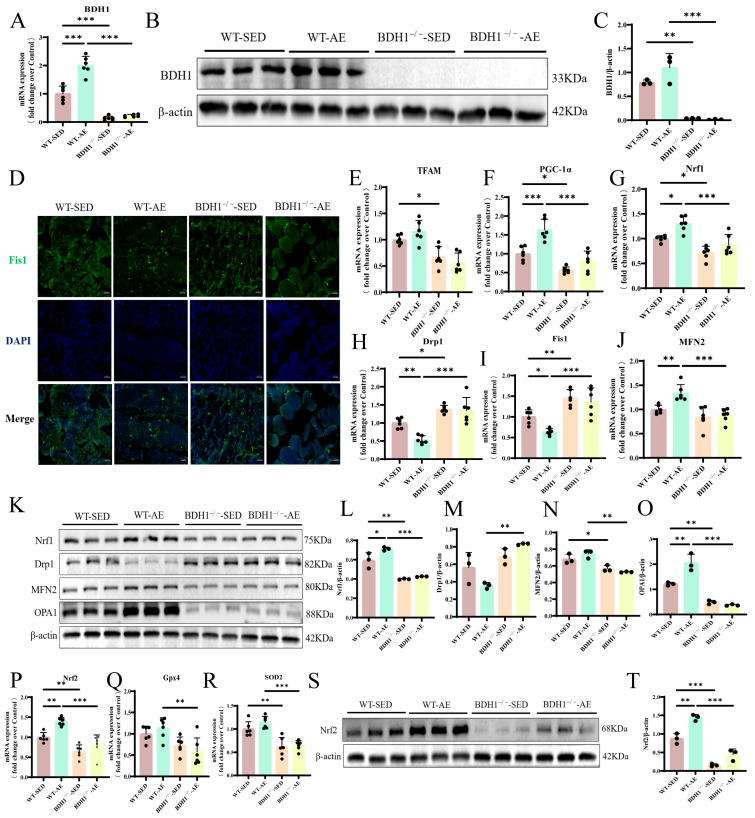
Effects of AE on MQC and oxidative stress in the gastrocnemius muscle of BDH1^−/−^ T2DM mice. (**A**). BDH1 mRNA expression level in the gastrocnemius muscle. *n* = 6. (**B**). Protein expression of BDH1 and internal control β-actin in the gastrocnemius muscle. (**C**). BDH1 protein level quantitative analysis. *n* = 3. (**D**). Representative images of Fis1 immunofluorescence staining in the gastrocnemius muscle. Scale bars = 20 μm. (**E**–**G**). mRNA expression levels of genes regulating mitochondrial biogenesis (TFAM, PGC-1α, and Nrf1) in the gastrocnemius muscle. *n* = 6. (**H**,**I**). mRNA expression levels of genes regulating mitochondrial fission (Drp1 and Fis1) in the gastrocnemius muscle. *n* = 6. (**J**). mRNA expression level of the gene regulating mitochondrial fusion (MFN2) in the gastrocnemius muscle. *n* = 6. (**K**). Protein expression of Nrf1, Drp1, MFN2, OPA1, and internal control β-actin in the gastrocnemius muscle. (**L**). Nrf1 protein level quantitative analysis. *n* = 3. (**M**). Drp1 protein level quantitative analysis. *n* = 3. (**N**). MFN2 protein level quantitative analysis. *n* = 3. (**O**). OPA1 protein level quantitative analysis. *n* = 3. (**P**–**R**). Antioxidant (Nrf2, Gpx4, SOD2) mRNA expression levels in the gastrocnemius muscle. *n* = 6. (**S**). Nrf2 and internal control β-actin protein expressions in the gastrocnemius muscle. (**T**). Nrf2 protein level quantitative analysis. *n* = 3. * *p* < 0.05, ** *p* < 0.01, *** *p* < 0.001. Original images of (**B**,**K**,**S**) can be found in Supplementary Materials.

We generated BDH1^−/−^ mice to further clarify the role of BDH1 in improving skeletal muscle MQC and oxidative stress in T2DM mice. Genotypic and protein analyses confirmed efficient BDH1 ablation (Figure 5A–C). On this basis, we detected the gene/protein expression levels of key mediators regulating MQC. The results revealed that BDH1^−/−^ impaired the regulatory effects of AE on mitochondrial dynamics and homeostasis in T2DM skeletal muscles compared with those in WT T2DM mice (Figure 5E–O). Furthermore, the imbalance in MQC was more evident in the knockout group (Figure 5E–O). The immunofluorescence results also showed no significant difference in the expression of the mitochondrial fission protein Fis1 between the BDH1^−/−^-SED and BDH1^−/−^-AE mice (Figure 5D). We further detected oxidative stress markers closely associated with impaired MQC. The results showed that the antioxidant effect of 8-week AE on skeletal muscles was weaker for the BDH1^−/−^ T2DM mice than for the WT T2DM mice, as evidenced by the almost lack of up-regulation of antioxidants (Nrf2, Gpx4, and SOD2) (Figure 5P–T). The oxidative stress status in BDH1^−/−^-SED mice was more severe than that in WT-SED mice (Figure 5P–T). In summary, BDH1 plays an important mediating role in AE-induced improvements in MQC and oxidative stress in T2DM mouse skeletal muscles.

### 3.5. BDH1^−/−^ Attenuates the Beneficial Effects of Aerobic Exercise on Inflammation and Fibrosis in the Skeletal Muscles of T2DM Mice

**Figure 6:**
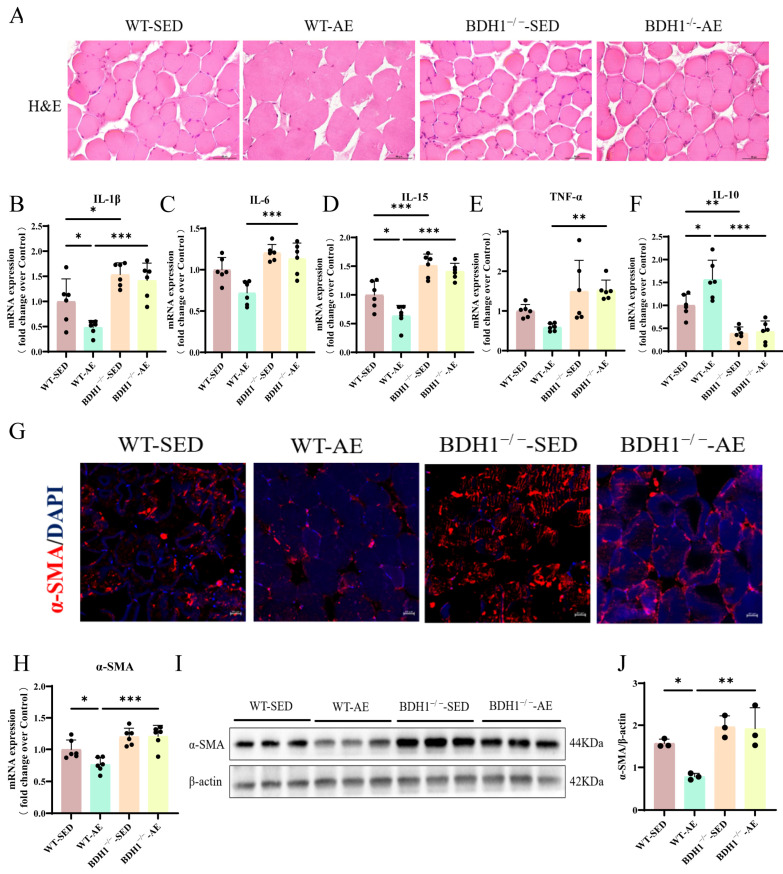
Effects of AE on inflammation and fibrosis in the gastrocnemius muscle of BDH1^−/−^ T2DM mice. (**A**). Representative images of H&E staining of the gastrocnemius muscle. Scale bars = 50 μm. (**B**–**E**). Pro-inflammatory cytokine (IL-1β, IL-6, IL-15, and TNF-α) mRNA expression levels in the gastrocnemius muscle. *n* = 6. (**F**). Anti-inflammatory cytokine IL-10 mRNA expression level in the gastrocnemius muscle. *n* = 6. (**G**). Representative images of α-SMA immunofluorescence staining in the gastrocnemius muscle. Scale bars = 20 μm. (**H**). α-SMA mRNA expression level in the gastrocnemius muscle. *n* = 6. (**I**). α-SMA and internal control β-actin protein expressions in the gastrocnemius muscle. (**J**). α-SMA protein level quantitative analysis. *n* = 3. * *p* < 0.05, ** *p* < 0.01, *** *p* < 0.001. Original images of (**I**) can be found in Supplementary Materials.

We also observed that AE reduced skeletal muscle inflammatory infiltration and narrowing the intermuscular space in WT-SED mice (Figure 6A). However, following BDH1^−/−^, AE induced almost no improvement in skeletal muscle inflammation or widened intermuscular spaces in T2DM mice (Figure 6A). Moreover, the histopathological damage in BDH1^−/−^-SED mice was more severe than that in WT-SED mice (Figure 6A). We detected the gene expression levels of pro-inflammatory mediators (IL-1β, IL-6, IL-15, and TNF-α) and anti-inflammatory factor IL-10. The results also showed analogous findings (Figure 6B–F). Finally, immunofluorescence, qRT-PCR, and Western blot analyses of α-SMA expression demonstrated that the beneficial effect of AE on the fibrotic marker α-SMA was attenuated in the knockout group, and the positive expression of α-SMA in BDH1^−/−^-SED mice was higher than that in WT-SED mice (Figure 6G–J). Overall, these results indicate that BDH1 is a crucial mediator for the AE-induced alleviation of skeletal muscle inflammatory infiltration and fibrosis in T2DM mice.

### 3.6. BDH1^−/−^ Attenuates the Beneficial Effects of Aerobic Exercise on Glycolipid Metabolism in the Skeletal Muscles of T2DM Mice

**Figure 7:**
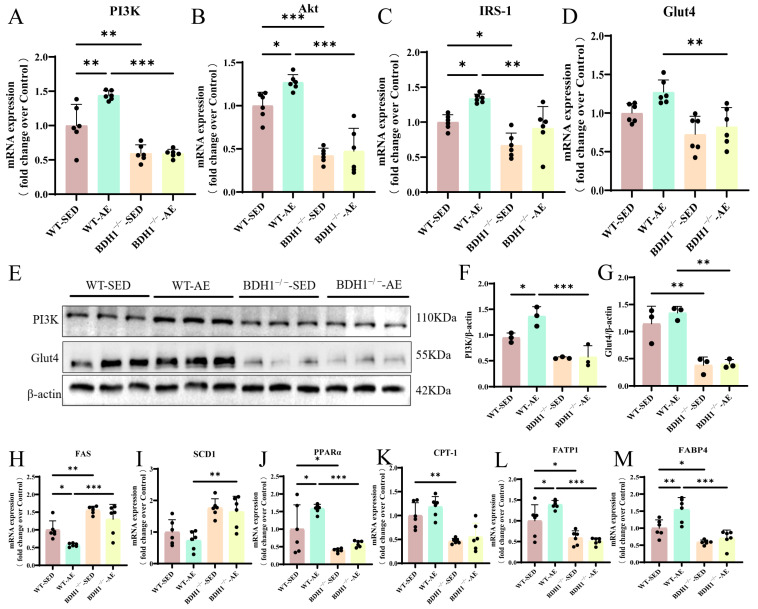
Effects of AE on glycolipid metabolism in the gastrocnemius muscle of BDH1^−/−^ T2DM mice. (**A**–**D**). mRNA expression levels of glucose metabolism-related indicators (PI3K, Akt, IRS-1, and Glut4) in the gastrocnemius muscle. *n* = 6. (**E**). Protein expression levels of PI3K, Glut4, and internal control β-actin in the gastrocnemius muscle. (**F**). PI3K protein level quantitative analysis. *n* = 3. (**G**). Glut4 protein level quantitative analysis. *n* = 3. (**H**–**M**). mRNA expression levels of lipid metabolism-related indicators (FAS, SCD1, PPARα, CPT-1, FATP1, and FABP4) in the gastrocnemius muscle. *n* = 6. * *p* < 0.05, ** *p* < 0.01, *** *p* < 0.001. Original images of (**E**) can be found in Supplementary Materials.

Detection of the expression levels of glucose metabolism-related indicators (PI3K, Akt, Glut4, and IRS-1) (Figure 7A–G) and lipid metabolism-related indicators (FAS, SCD1, PPARα, CPT-1, FATP1, and FABP4) (Figure 7H–M) revealed that the beneficial effect of AE on glycolipid metabolism in T2DM skeletal muscle was less marked in the knockout group than in the WT T2DM mice (Figure 7A–M). Furthermore, the impairment of glycolipid metabolism in BDH1^−/−^-SED mice was more severe than that in WT-SED mice (Figure 7A–M). Overall, these findings suggest that BDH1 mediates the protective role of AE in maintaining glycolipid metabolic and mitochondrial homeostasis in T2DM mouse skeletal muscles.

## 4. Discussion

As a heterogeneous metabolic disease, T2DM is characterized by metabolic and signaling dysregulation [33,34]. It exhibits systemic metabolic disorders: Elevated circulating free fatty acids inhibit the stimulatory effect of insulin secretion on glucose uptake in skeletal muscles and the negative regulation of hepatic gluconeogenesis. This phenomenon results in increased circulating glucose concentrations, imbalanced endocrine regulation of metabolism, and impaired immune effector function [35]. Multiple physiological stimuli can alter metabolic remodeling. Among these interventions, exercise therapy has emerged as a key metabolic regulatory strategy for T2DM risk management because of its accessibility and cost-effectiveness [36].

### 4.1. Metabolic Remodeling in T2DM Skeletal Muscles

T2DM is closely associated with the imbalance of metabolic homeostasis of energy substrates such as carbohydrates, amino acids, and lipids [37]. Hyperglycemia and lipotoxicity are two major risk factors mediating the development and progression of T2DM [38]. Excessive accumulation of free fatty acids also inhibits glucose utilization efficiency in skeletal muscles, thereby triggering insulin resistance [37]. Stable mitochondrial function is a prerequisite for ensuring skeletal muscle metabolic plasticity and is responsible for meeting the energy metabolic demands of cells throughout the body [39]. Therefore, impaired mitochondrial network dynamics in skeletal muscle is closely associated with glucolipotoxic obesity, which involves insulin resistance and lipotoxicity accumulation. Hyperglycemia induces mitochondrial fragmentation in the skeletal muscle of patients with T2DM by triggering Drp1-mediated mitochondrial fission or impaired mitochondrial fusion. Sustained mitochondrial fragmentation exacerbates ROS release, thereby aggravating oxidative stress and impaired insulin signaling [40]. Mitochondrial biogenesis is the process of generating new mitochondria from existing ones in response to the body’s high energy demands under stress conditions. Increased functional mitochondria can enhance ATP production efficiency and inhibit oxidative stress, thereby protecting the stable output of insulin signaling pathways [41]. Chronic systemic inflammation is also one of the pathological mechanisms inducing insulin resistance and β-cell dysfunction and is closely associated with T2DM development [16]. Diabetes-induced inflammatory infiltration impairs anabolic efficiency and reduces muscle blood supply, ultimately triggering muscle fibrosis [42].

### 4.2. Aerobic Exercise Improves Metabolic Remodeling in T2DM Skeletal Muscles

Physical activity improves insulin sensitivity by increasing Glut4 protein content, enhancing skeletal muscle glucose uptake, regulating intracellular lipid redistribution, and reducing muscle lipid accumulation [43]. Wang et al. [44] demonstrated that 30 weeks of HFD impaired glucose tolerance and lipid metabolic homeostasis in mice, and 8 weeks of AE could promote energy metabolism. Hu et al. [45] found that HFD up-regulated SCD1 in mouse skeletal muscles, fasting blood glucose, and lipid levels, while 6 weeks of moderate-intensity AE effectively alleviated glycolipid metabolic disorders. Consistent with these findings, our study also demonstrated that 8 weeks of AE exerted a beneficial regulatory effect on glycolipid metabolism in mice. A recent work demonstrated that 8-week AE alleviates mitochondrial network dynamic imbalance associated with abnormal glycemic control by inducing MFN2 expression in the gastrocnemius muscle of diabetic mice [46]. Lin et al. [47] found that 8-week AE in rats reduced the expression of mitochondrial fission proteins Fis1 and Drp1 and up-regulated the content of MFN2 in the soleus muscle, with the underlying mechanism involving activation of the irisin/AMP-activated protein kinase pathway. Previous findings from our team also confirmed that resistance exercise promotes mitochondrial biogenesis in T2DM mice by up-regulating the Nrf1/mtDNA signaling axis [48]. Similarly, Wang et al. [44] found that 8-week AE reversed the down-regulation of PGC-1α and MFN2 in the skeletal muscles of mice with impaired glucose tolerance and increased the levels of antioxidant enzymes in the blood. Consistent with these reports, our study revealed that the same duration of AE in T2DM mice improved mitochondrial homeostasis and peroxidation status. In terms of exercise against inflammation and fibrosis, Li et al. [49] demonstrated that AE alleviated chronic inflammation associated with insulin resistance by regulating the levels of IL-1β and IL-10 in skeletal muscles. Six weeks of AE also inhibited transforming growth factor-β signaling in the gastrocnemius muscle of T2DM mice and promoted extracellular matrix remodeling and muscle regeneration, thereby mitigating muscle fibrosis [50]. Similar to the significant beneficial effects of swimming exercise on pulmonary inflammation and fibrosis improvement in T2DM mice previously reported by our team, our present findings revealed that 8-week AE suppressed the inflammatory infiltration and profibrotic state in T2DM skeletal muscles [51].

### 4.3. BDH1^−/−^ Attenuates the Beneficial Effect of Aerobic Exercise on Metabolic Remodeling in T2DM Skeletal Muscles

As a biological enzyme involved in hepatic glucose metabolism, BDH1 knockdown in mouse liver inhibits tricarboxylic acid cycle flux, pyruvate cycling rate, hepatic gluconeogenesis, and mitochondrial succinyl-CoA levels [52]. Exercise intervention increases BDH levels in the gastrocnemius muscle of rats by approximately threefold [22]. However, whether BDH1 plays a central role in AE-regulated glycolipid metabolic homeostasis in T2DM skeletal muscle remains unclear. In our study, BDH1 knockout attenuated the protective effects of AE on glycolipid metabolic disorders in T2DM skeletal muscles, and the impairment of energy metabolism in the gastrocnemius muscle of BDH1^−/−^ T2DM mice was more severe than that in WT T2DM mice. Existing studies have shown that BDH1 overexpression in cardiomyocytes reduces excessive mitochondrial ROS production and improves oxidative stress and cardiac remodeling associated with heart failure [53]. Furthermore, BDH1 drives Nrf2 signaling in Raw264.7 cells by affecting fumarate metabolic flux, thereby inhibiting oxidative stress and ROS generation [54]. In diabetic nephropathy, BDH1 overexpression can further activate Nrf2 by promoting the metabolic flux composed of β-HB-acetoacetate-succinate-fumarate [55]. In delaying retinal degeneration under ischemic conditions, BDH1 overexpression induces the up-regulation of Nrf2, a key transcription factor that regulates antioxidant response elements, by mediating the enhancement in β-HB and fumarate metabolic flux [56]. Under coculture conditions, BDH1-overexpressing hTERT-immortalized fibroblasts increased mitochondrial quality and the expression of the mitochondrial biogenesis marker heat shock protein 60 in human breast cancer MCF7 cells [57]. Therefore, we further verified the protective role of BDH1 in AE-induced improvements in MQC and oxidative stress. Xu et al. [58] showed that excessive ROS release in BDH1-knockdown LO2 cells exacerbated the pro-secretory effects of palmitic acid on inflammatory factors IL-1β and IL-18, whereas adeno-associated virus-mediated BDH1 overexpression effectively alleviated hepatic fibrosis and inflammation in diabetic mice. The underlying mechanism also involves BDH1 further activating Nrf2 by promoting the metabolic flux composed of β-HB-acetoacetate-succinate-fumarate, thereby inhibiting the driving effect of palmitic acid lipotoxicity on ROS release [58]. Another study indicated that silencing BDH1 in high glucose-induced RAW264.7 cells promoted excessive ROS production and stimulated the secretion of macrophage inflammatory factors such as IL-1β, IL-18, and TNF-α [54]. Wan et al. [55] found that BDH1 overexpression antagonized the secretion of IL-1β and IL-18 induced by high glucose or palmitic acid. In addition, in vivo BDH1 overexpression alleviated renal fibrosis, suggesting that BDH1 may be a potential therapeutic target for diabetic kidney disease. A study on lung cancer cell proliferation and migration revealed that BDH1 down-regulation inhibited the activation of the AMP-activated protein kinase-mammalian target of rapamycin autophagy signaling pathway [59]. In the inhibition of macrophage ferroptosis, BDH1 mediates Nrf2 activation [60]. AMP-activated protein kinase and Nrf2 have critical regulatory effects in mediating exercise responses, and skeletal muscles also exhibit metabolic heterogeneity and flexible energy regulation [61,62]. Building upon this, our study further examined whether BDH1 mediates AE-induced improvements in inflammation and fibrosis in T2DM skeletal muscle.

Our study is the first to verify that 8 weeks of AE improves glycolipid metabolism and MQC by up-regulating BDH1, thereby alleviating oxidative stress, inflammation, and fibrosis in the skeletal muscles of T2DM. The dynamic properties of mitochondria, such as the balance between fusion and fission, ensure the efficient transmission of mitochondrial energy and high-performance oxidative phosphorylation (OXPHOS) [63]. Although the exchange of mitochondrial contents and the degradation of damaged mitochondrial components promote the homogenization of the mitochondrial population, this study lacks the detection of indicators related to mitochondrial OXPHOS and other pathways involved in mitochondrial energy metabolism. Future supplementation with data on respiratory complex activity will comprehensively strengthen the evidence for the beneficial effect of BDH1 on mitochondrial function during AE. Sadeesh et al. focused on amino acid metabolism and further revealed tissue-specific differences in mitochondrial function, laying the foundation for the heterogeneity of mitochondrial bioenergetics in different T2DM tissues [64]. Owing to the tissue-specific expression profiles of nucleus-encoded mitochondrial genes, BDH1-mediated ketone body metabolism and utilization may exhibit substantial differences between T2DM skeletal muscles and other tissues [64]. While verifying the tissue specificity of nucleus-encoded mitochondrial gene expression, KEGG pathway analysis also demonstrated tissue-specific energy metabolism regulatory strategies involving metabolic pathways that participate in the regulation of mitochondrial ATP synthesis. In particular, skeletal muscles and other insulin-sensitive tissues possess distinct mitochondrial bioenergetic regulatory mechanisms; among which, BDH1 may mediate AE to activate the skeletal muscle-specific ATP synthesis pathway, thereby exerting a bioenergetic regulatory function [65]. Moreover, the differential expression of mitochondrial protein-coding genes implies that mitochondria in different tissues exhibit specificity in terms of energy production and substrate utilization. For instance, muscle and brain tissues tend to have relatively high mitochondrial DNA copy numbers, which meet their high energy demands and induce strong dependence on OXPHOS [66]. Therefore, whether the regulatory effect of BDH1 on metabolic remodeling in T2DM skeletal muscles during AE involves changes in the subunit activity of OXPHOS complexes may warrant further investigation.

### 4.4. Limitations and Perspectives

As a key enzyme catalyzing the NAD^+^/NADH-coupled interconversion between acetoacetate and β-HB, BDH1 plays a central role in regulating ketone body metabolism [54]. Our team previously measured blood ketone body levels in WT T2DM mice following a single bout of exercise (15 m/min, 60 min) and found that the levels peaked at 60 min post-exercise (1.92 ± 0.78 mM) and then gradually decreased until returning to baseline levels at 240 min post-exercise. After 8 weeks of exercise intervention, ELISA was further employed to detect the serum β-HB abundance. No significant fluctuations were observed among the WT-CON, WT-SED and WT-AE groups. This finding suggests that although AE can acutely elevate circulating β-HB levels in mice, the short-term accumulated β-HB will be metabolized by peripheral tissues, and long-term exercise intervention will not cause an increase in baseline β-HB levels [27]. Therefore, future studies are highly necessary to further explore whether ketone bodies, as exercise mimetics, act as energy substrates or signaling molecules in improving the pathological phenotype of T2DM skeletal muscles [67]. Systemic BDH1 knockout models involve all tissues and cells in the body that may express BDH1, making it difficult to distinguish between the effect of liver-derived ketone body supply and the intrinsic role of BDH1 in skeletal muscles. Furthermore, BDH1 knockout in other tissues may further interfere with the skeletal muscle phenotype through interorgan crosstalk. Therefore, the lack of skeletal muscle-specific BDH1 knockout models is a crucial direction that must be addressed in future research. Furthermore, introducing C2C12 cells or primary myocytes, utilizing small interfering RNA-mediated gene silencing strategies, and combining with β-HB or its analogs treatment will optimize the evidence chain supporting BDH1 as a therapeutic target for the AE-induced improvement in metabolic remodeling in myocytes under T2DM-mimetic conditions. To verify the protective effect of BDH1-mediated AE on skeletal muscle in T2DM, we only employed knockout models for validation. In the future, supplementing any auxiliary experiments involving BDH1-specific overexpression or exogenous β-HB administration will strengthen the demonstration of BDH1-mediated regulatory effects of exercise. Moreover, only the total protein expression of Glut4 was detected in this study. Its change is more indicative of the molecular regulatory status of the insulin signaling pathway rather than glucose uptake function. The relevant metabolic functions still need to be further verified by Glut4 plasma membrane translocation assays or functional experiments. This study mainly focused on the effect of BDH1^−/−^ on metabolic remodeling in T2DM skeletal muscles. Given that body weight and blood glucose were not systematically monitored under the knockout condition, the comprehensive evaluation of systemic metabolic effects may be limited. In future studies, relevant indicators will be supplemented to improve the phenotypic analysis of mice. Finally, in addition to insulin resistance, T2DM is characterized by the dysfunction of β-cells (insulin-secreting units) [2], such as reduced mass caused by an imbalance between β-cell renewal and apoptosis [68]. BDH1 mediates responses to energy sensing during AE, which may also affect the insulin secretion or survival status of β-cells. However, this study did not assess changes in BDH1 activity in β-cells. Therefore, supplementary approaches such as insulin secretion assays and β-cell apoptosis detection will clarify whether the improvement in islet function contributes to the metabolic protective effect of BDH1 on T2DM skeletal muscles during AE.

## 5. Conclusions

This study revealed that 8-week AE can up-regulate BDH1 and improve glycolipid metabolism disorders, MQC imbalance, inflammation, oxidative stress, and fibrosis in the gastrocnemius muscle of T2DM mice, thereby inducing metabolic remodeling. Compared with that of WT T2DM mice, the gastrocnemius muscle of BDH1^−/−^ T2DM mice exhibited severe imbalance in metabolic remodeling, and BDH1^−/−^ attenuated the aforementioned beneficial effects of AE on the skeletal muscles of T2DM mice. These results suggest that BDH1 may play a crucial mediating role in the AE-induced improvement in skeletal muscle metabolic remodeling in T2DM. Given that BDH1 serves as a potential molecular target mediating the improvement in skeletal muscle metabolic phenotypes in T2DM via AE, this may provide a new direction for future targeted intervention research on skeletal muscle metabolic remodeling in diabetes.

## Figures and Tables

**Table 1:** Primer sequences for qRT-PCR analysis.

Gene	Forward Primer	Reverse Primer
IL-1β	GAAATGCCACCTTTTGACAGTG	TGGATGCTCTCATCAGGACAG
IL-6	TAGTCCTTCCTACCCCAATTTCC	TTGGTCCTTAGCCACTCCTTC
IL-15	CATCCATCTCGTGCTACTTGTG	GCCTCTGTTTTAGGGAGACCT
TNF-α	CCTGTAGCCCACGTCGTAG	GGGAGTAGACAAGGTACAACCC
IL-10	CTTACTGACTGGCATGAGGATCA	GCAGCTCTAGGAGCATGTGG
α-SMA	GTCCCAGACATCAGGGAGTAA	TCGGATACTTCAGCGTCAGGA
TFAM	AACACCCAGATGCAAAACTTTCA	GACTTGGAGTTAGCTGCTCTTT
PGC-1α	GAAAGGGCCAAACAGAGAGA	GTAAATCACACGGCGCTCTT
Nrf1	AGCACGGAGTGACCCAAAC	TGTACGTGGCTACATGGACCT
Drp1	ACTGATTCAATCCGTGATGAGT	GTAACCTATTCAGGGTCCTAGC
Fis1	AGAGCACGCAATTTGAATATGCC	ATAGTCCCGCTGTTCCTCTTT
MFN2	AGAACTGGACCCGGTTACCA	CACTTCGCTGATACCCCTGA
Nrf2	TCTTGGAGTAAGTCGAGAAGTGT	GTTGAAACTGAGCGAAAAAGGC
Gpx4	GAGGCAAGACCGAAGTAAACTA	CCGAACTGGTTACACGGGAA
SOD2	CCGTCCTCCCCTCCGCTGAT	ACGCCGCCCGACACAACATT
PI3K	ACCACGGTTTGGACTATGGAA	TACAGTAGTGGGCTTGGGTG
Akt	GCCGCTACTATGCCATGAAGATCC	GCAGGACACGGTTCTCAGTAAGC
IRS-1	GTCCTAGGGACACTCTTGACTAAC	TGCCCAAAGGAAAGACAGGATAAA
Glut4	GATGTGCCGTCGGGTTTCCAGCA	TGAGGGGTTGCCTTGTGGGATGG
FAS	TATCAAGGAGGCCCATTTTGC	TGTTTCCACTTCTAAACCATGCT
SCD1	CCAGAGGAGGTACTACAAGCC	GCACCAGAGTGTATCGCAAG
PPARα	TACTGCCGTTTTCACAAGTGC	AGGTCGTGTTCACAGGTAAGA
CPT-1	TGGCATCATCACTGGTGTGTT	CACCATAGCCGTCATCAGCAAC
FATP1	GGCTCCTGCGGCTTCAACAG	GTCTAGGGTCCGATTGATCTTTG
FABP4	AAGGTGAAGAGCATCATAACCCT	TCACGCCTTTCATAACACATTCC
BDH1	CACCGGAGTGTGTGTAAGGC	CTCGTCTGAACCCGTAGCTC
β-actin	CGTGCGTGACATCAAAGAGAA	GCTCGTTGCCAATAGTGATGA

## Data Availability

The original contributions presented in this study are included in this article; further inquiries can be directed to the corresponding authors.

## Supplementary Materials

The following supporting information can be downloaded at https://www.mdpi.com/article/10.3390/biom16010115/s1, Full Original western blot images are provided in supplementary figures.
