# Supplementary material for: BDH1 Mediates Aerobic Exercise-Induced Improvement in Skeletal Muscle Metabolic Remodeling in Type 2 Diabetes Mellitus
Source: Biomolecules. 2026 Jan 8;16(1):115. doi: 10.3390/biom16010115 (PMC12839089; doi:10.3390/biom16010115)

Figure S2H:

Western blots of Nrf1:

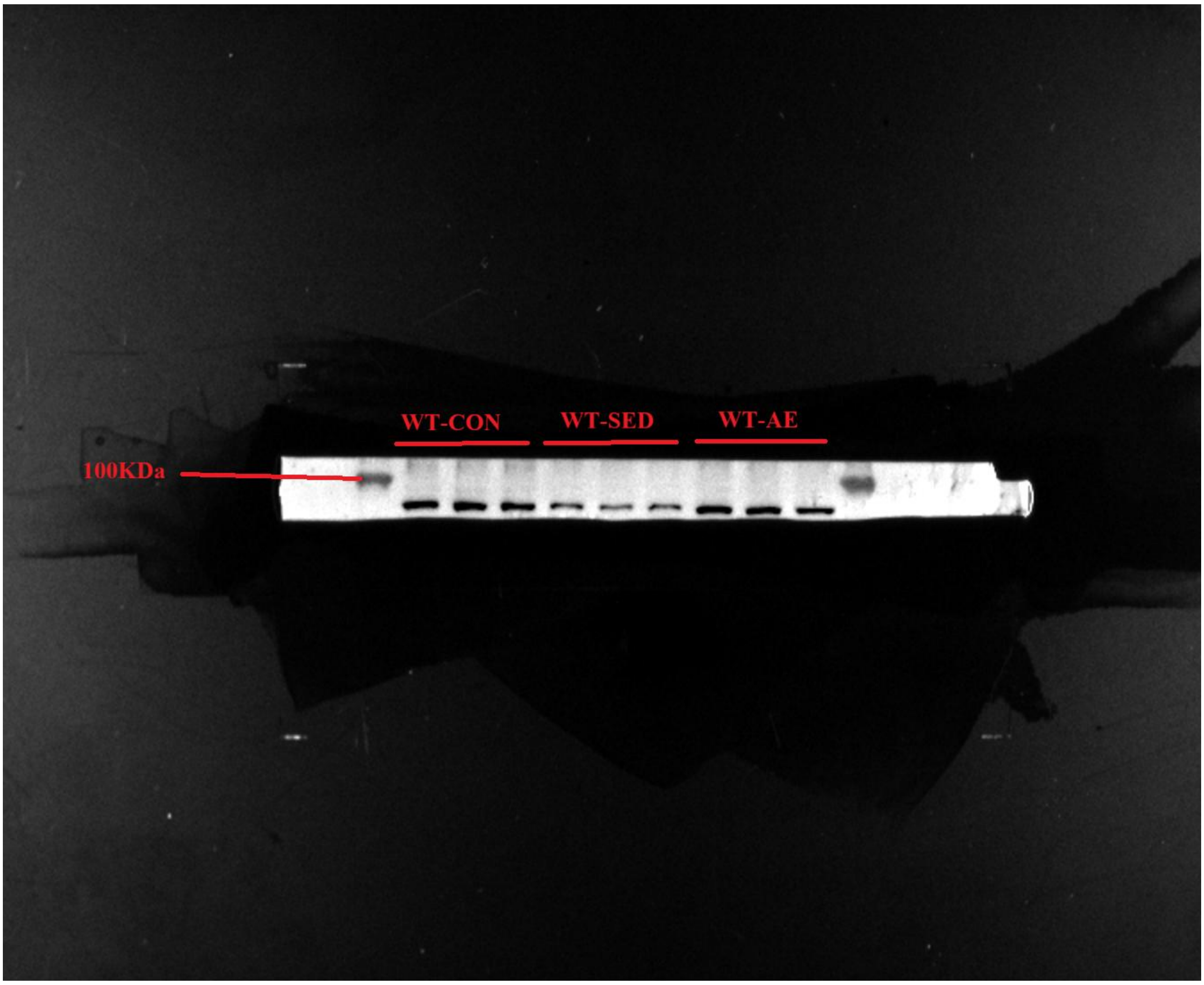

Western blots of Drp1:

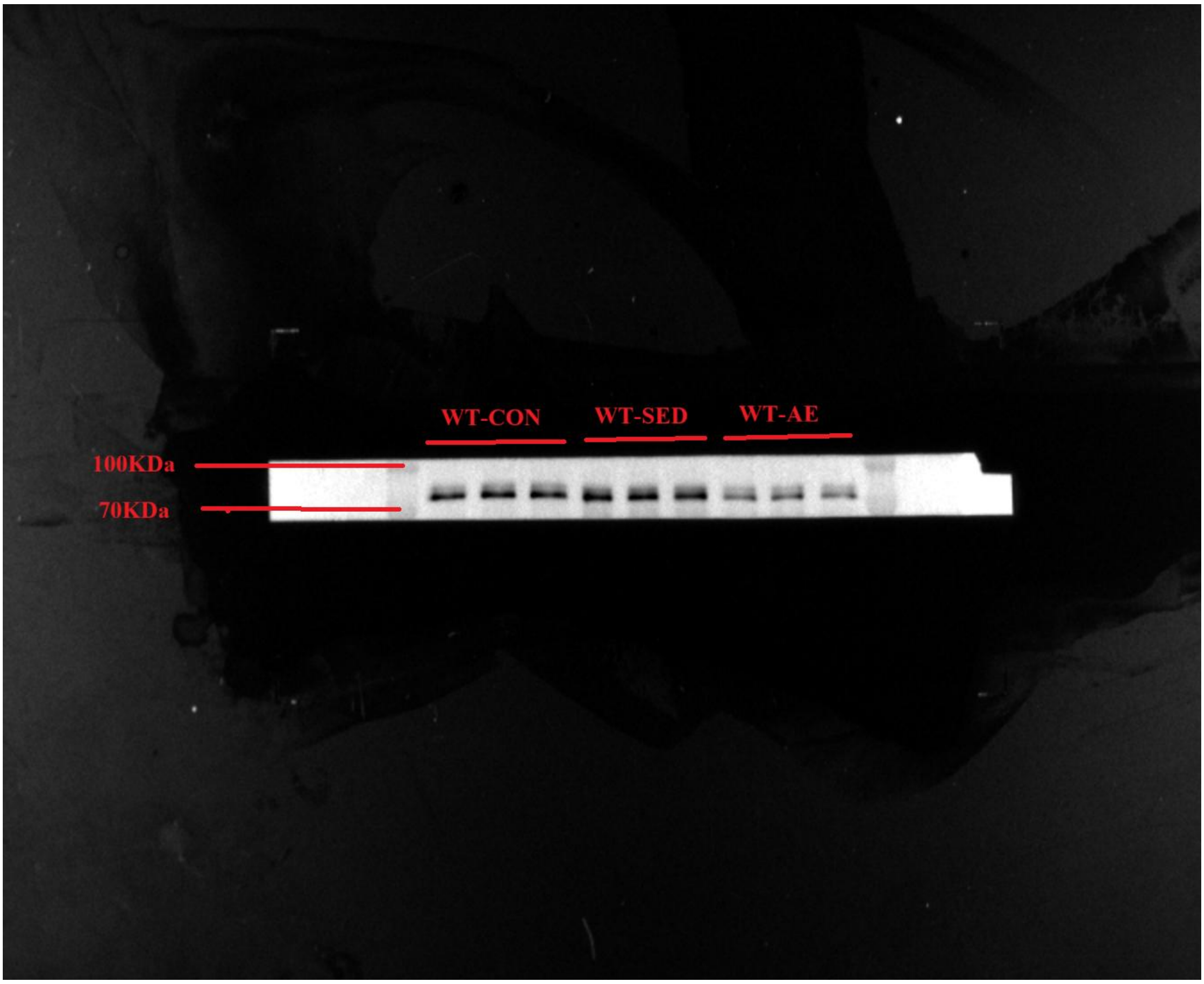

Western blots of MFN2:

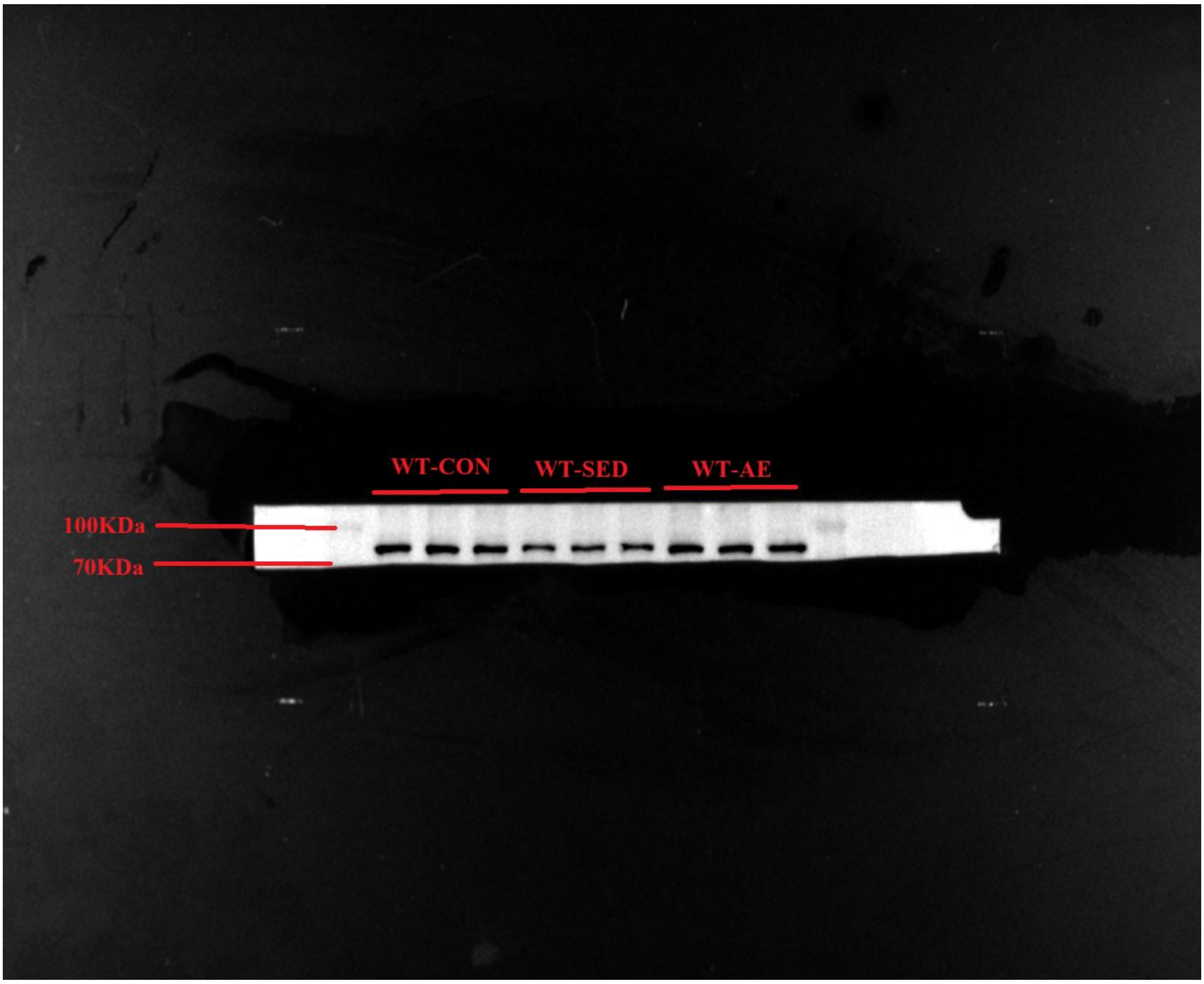

Western blots of OPA1:

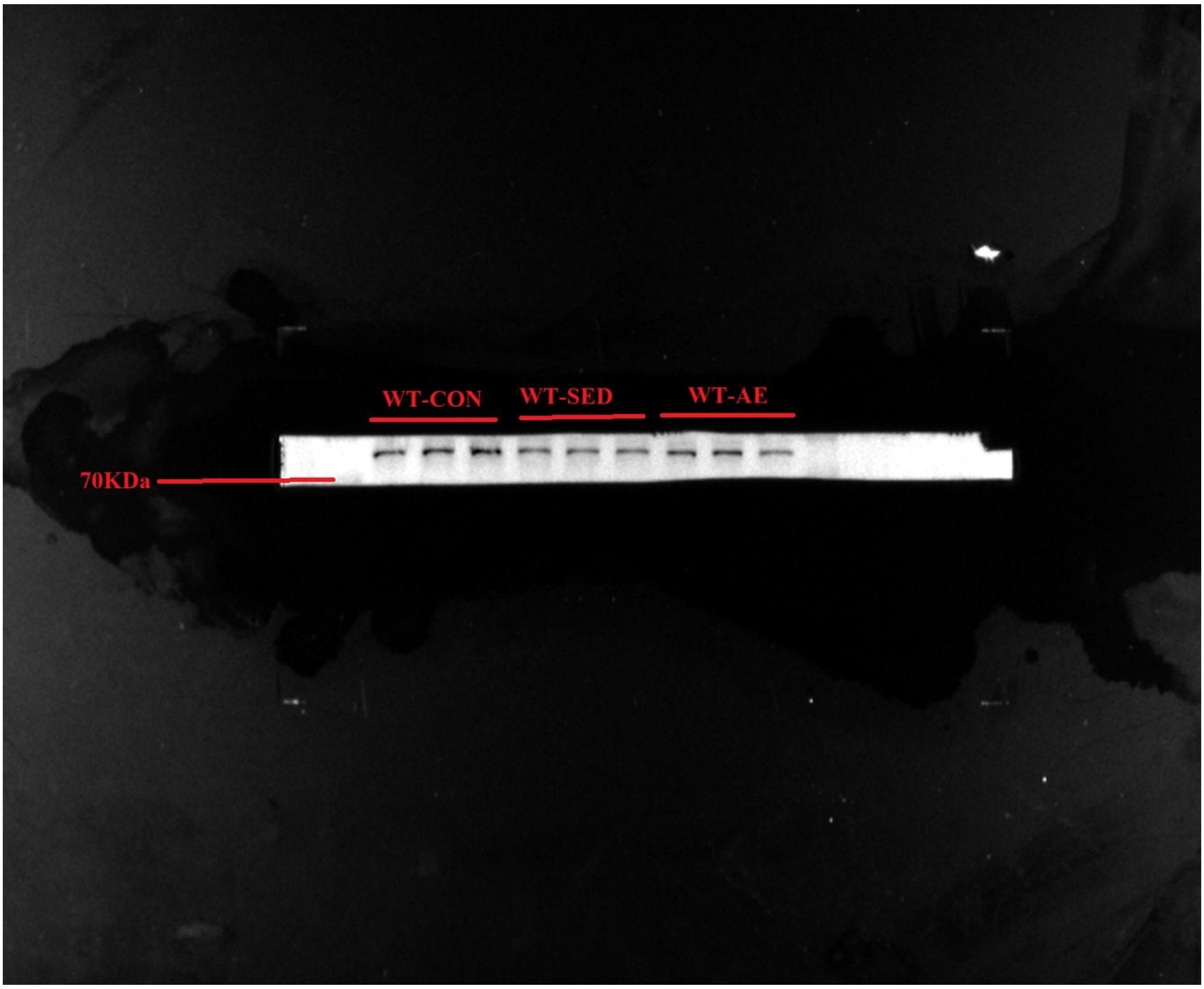

Western blots of β-actin:

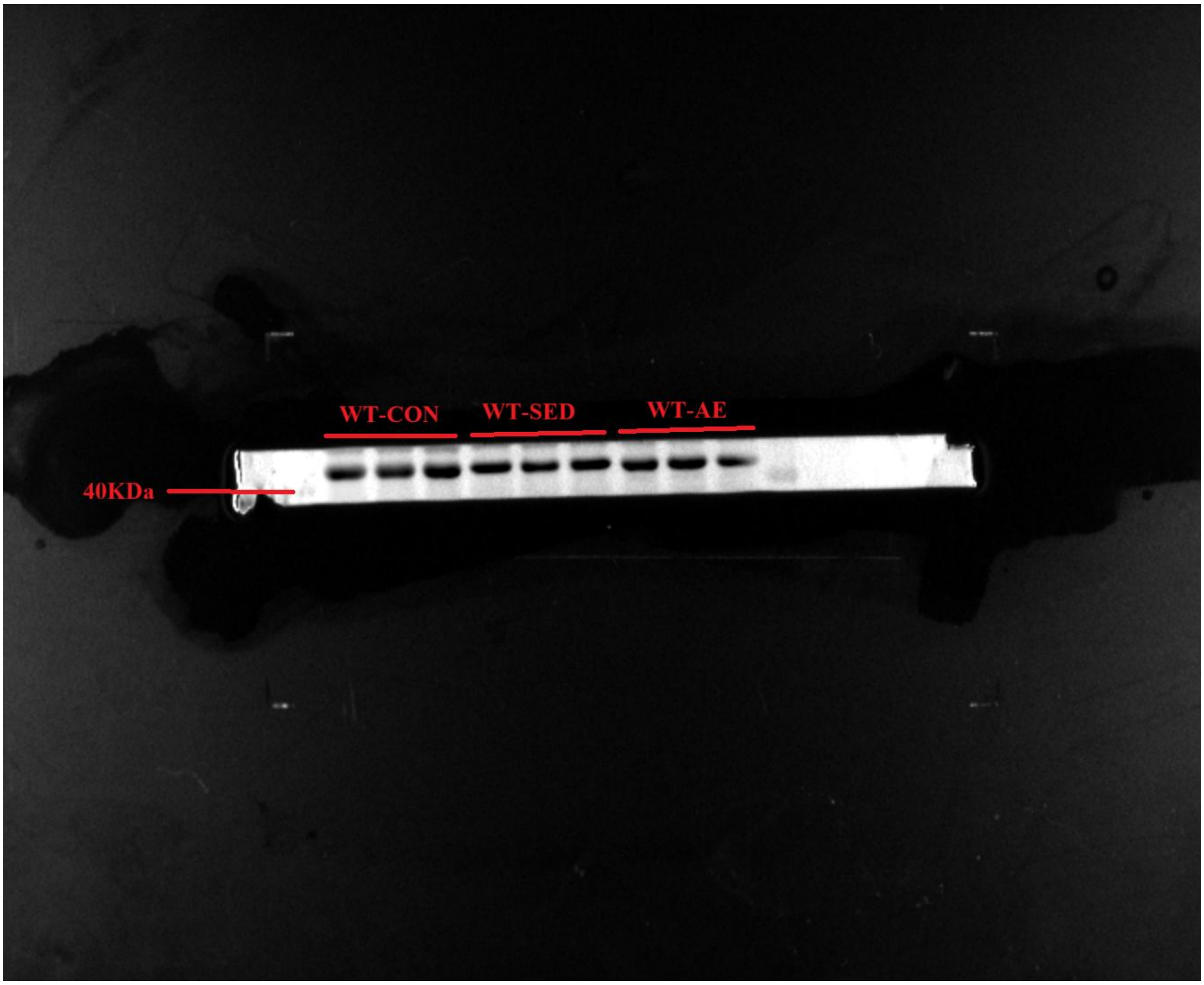

Figure S2P:

Western blots of Nrf2:

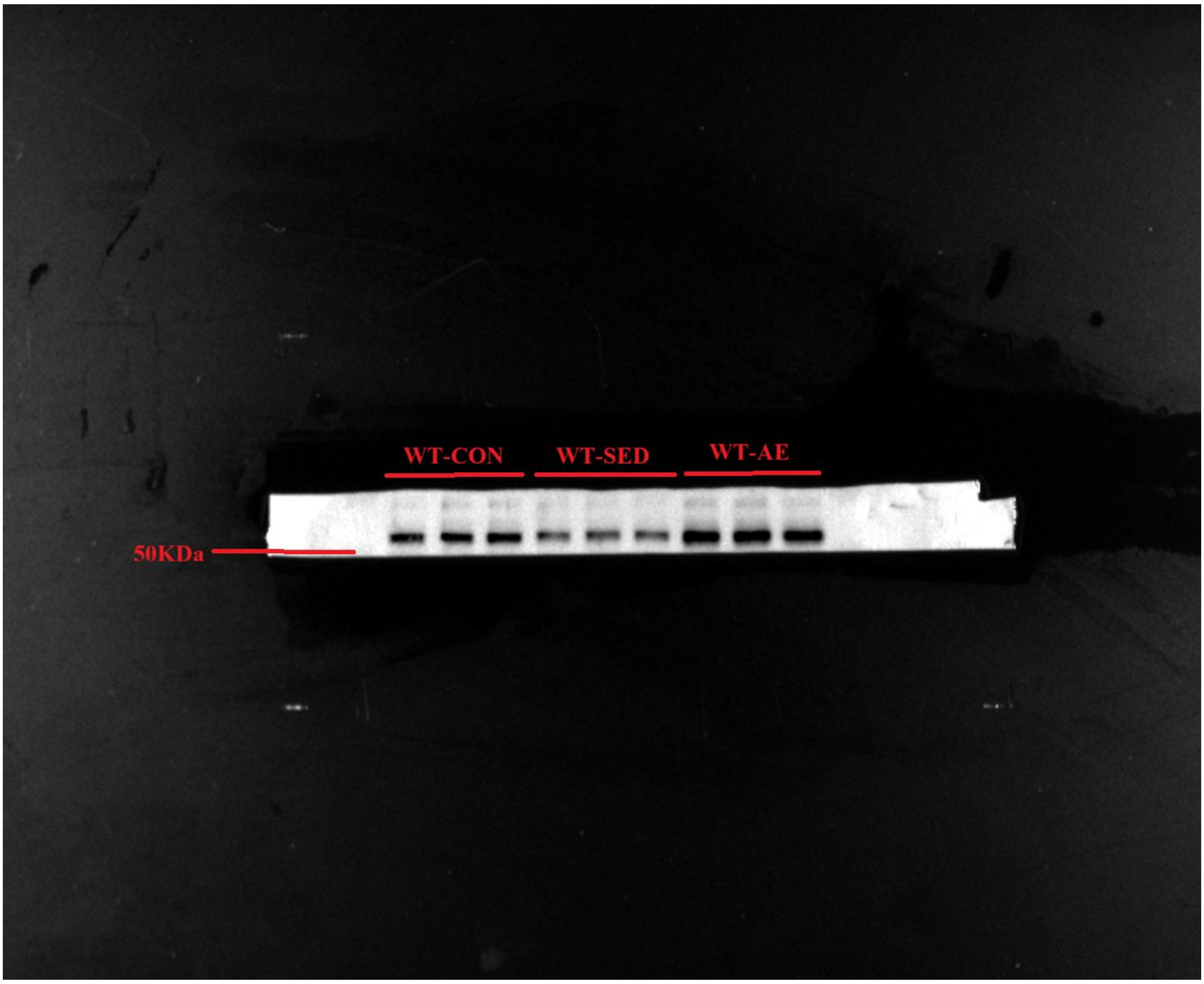

Western blots of β-actin:

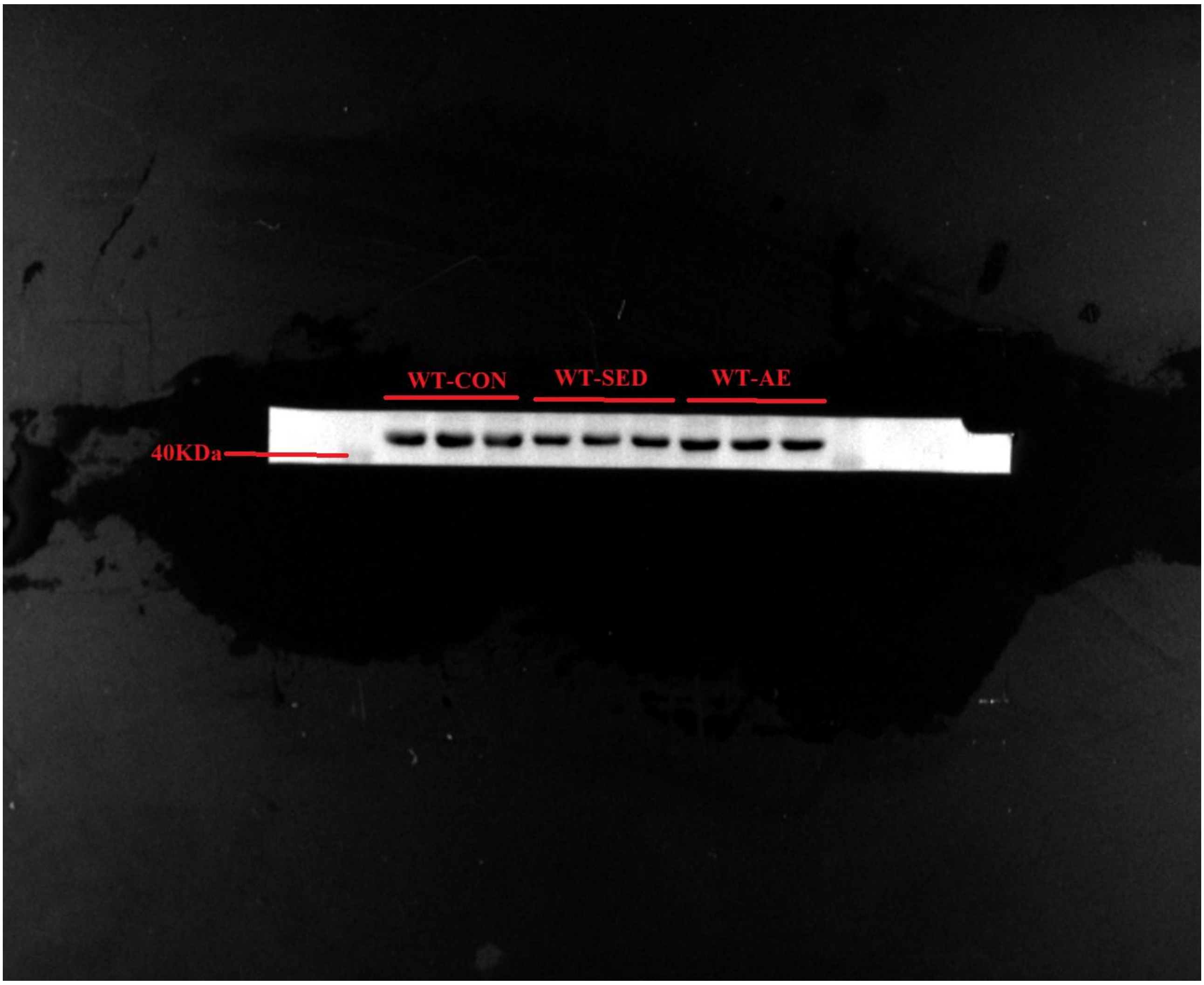

Figure S3H:

Western blots of α-SMA:

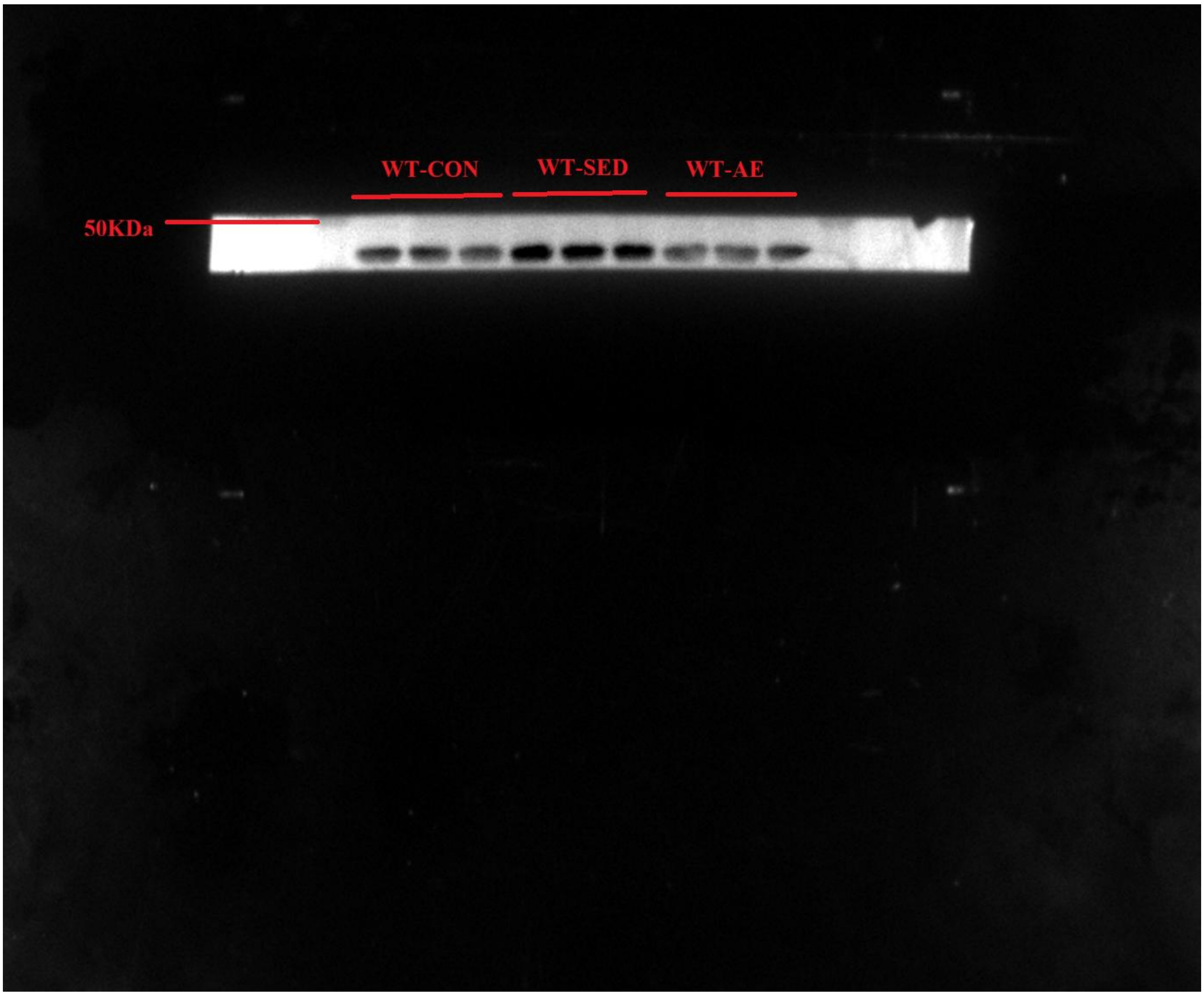

Western blots of β-actin:

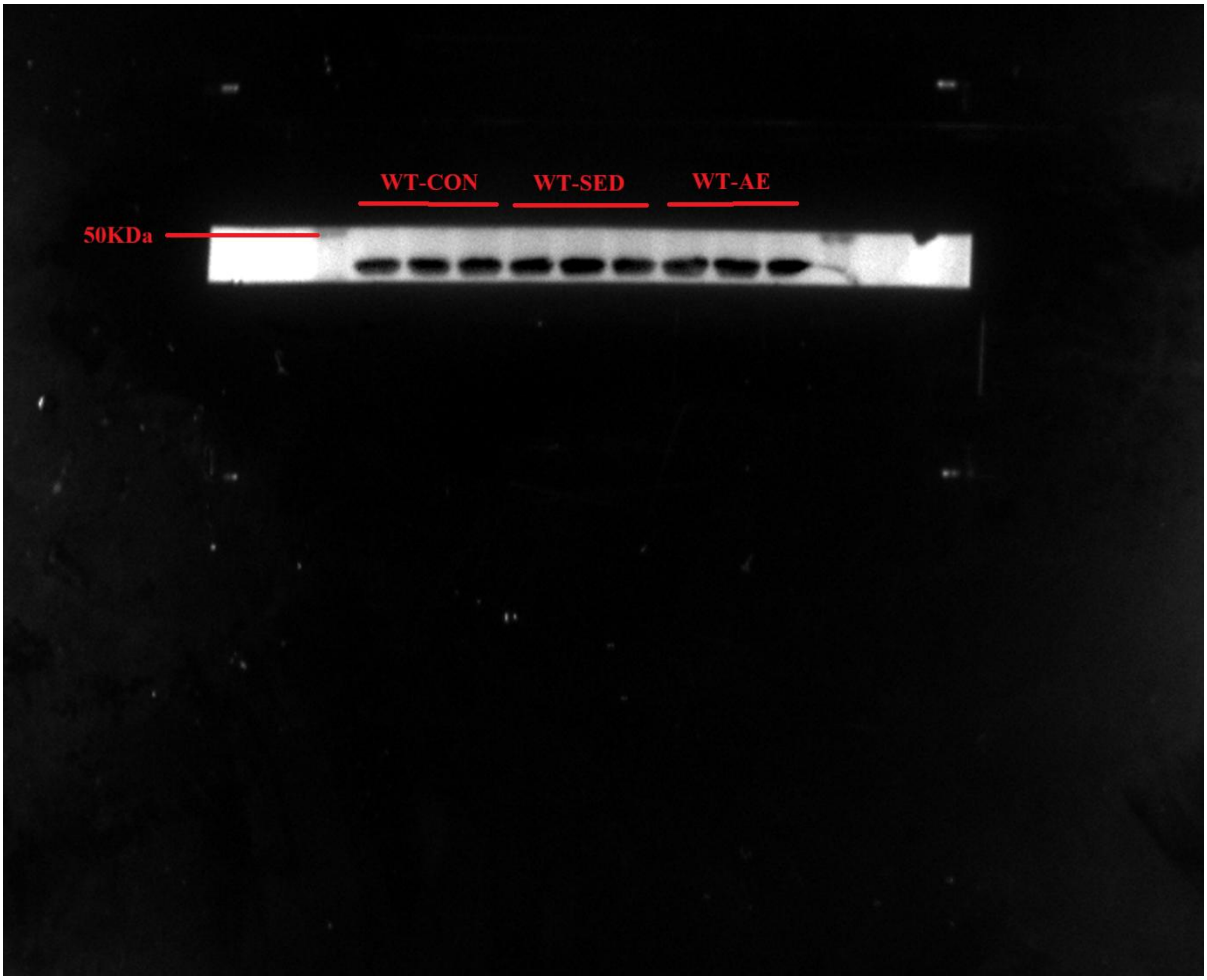

Figure S4E:

Western blots of PI3K:

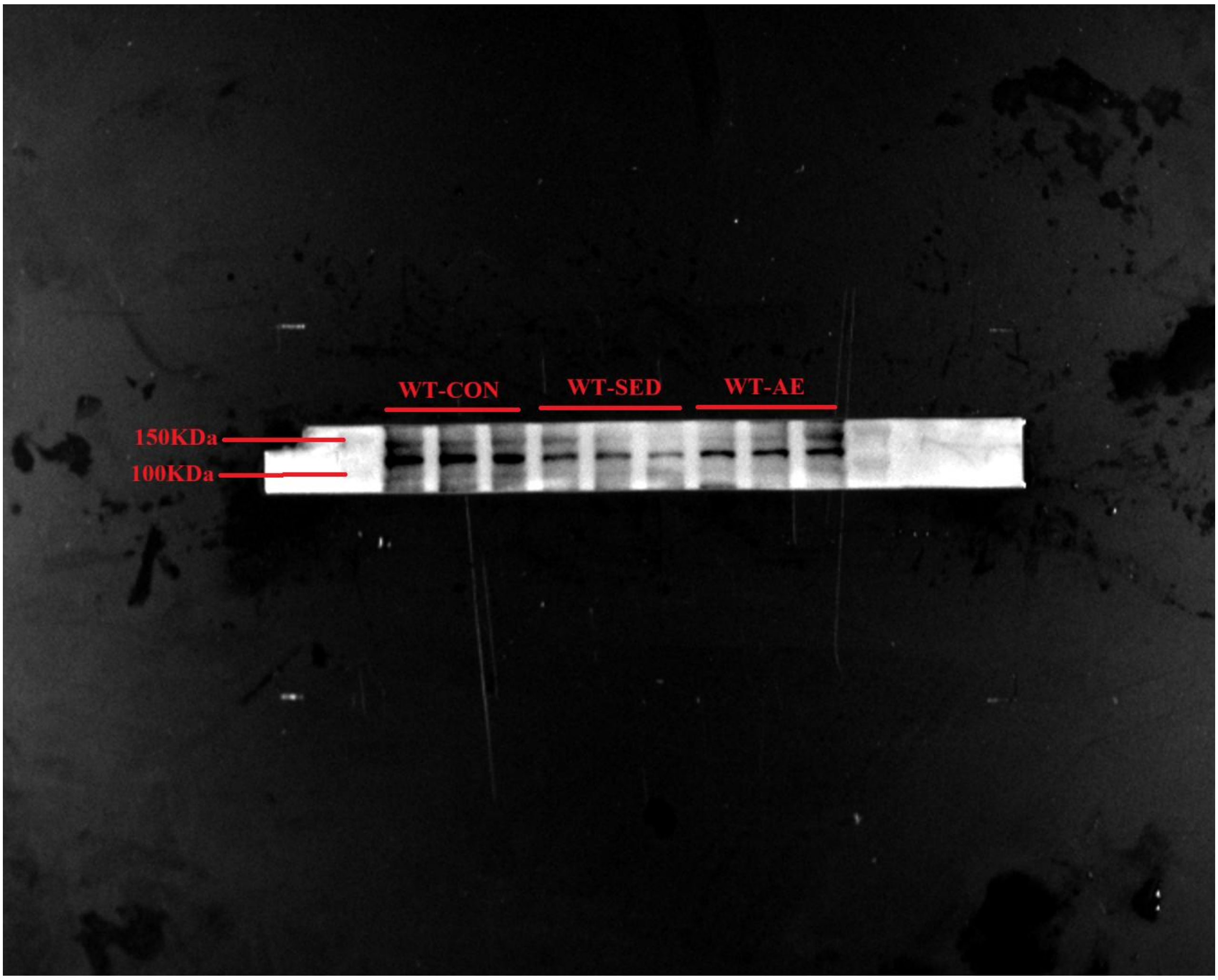

Western blots of p-Akt:

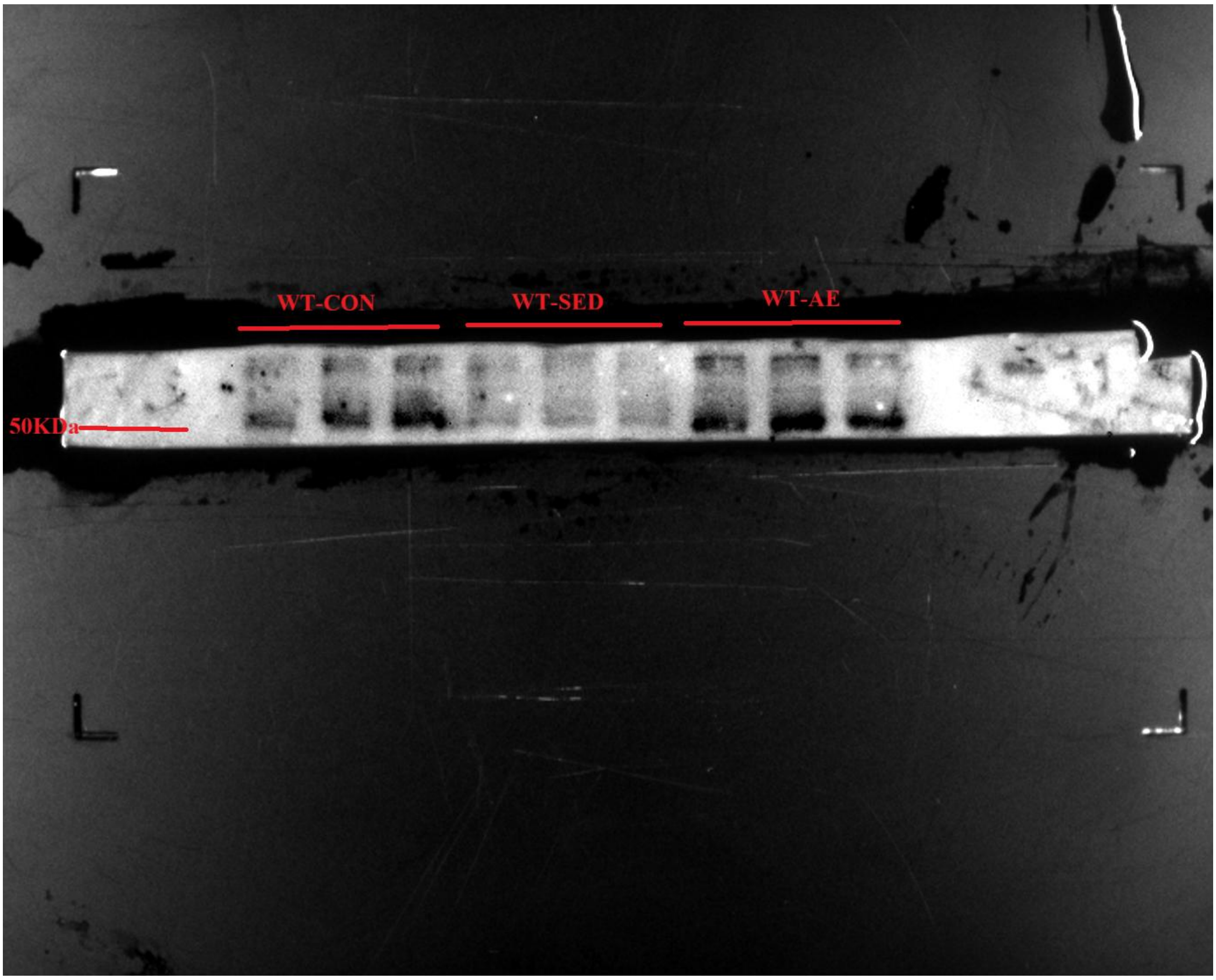

Western blots of Glut4:

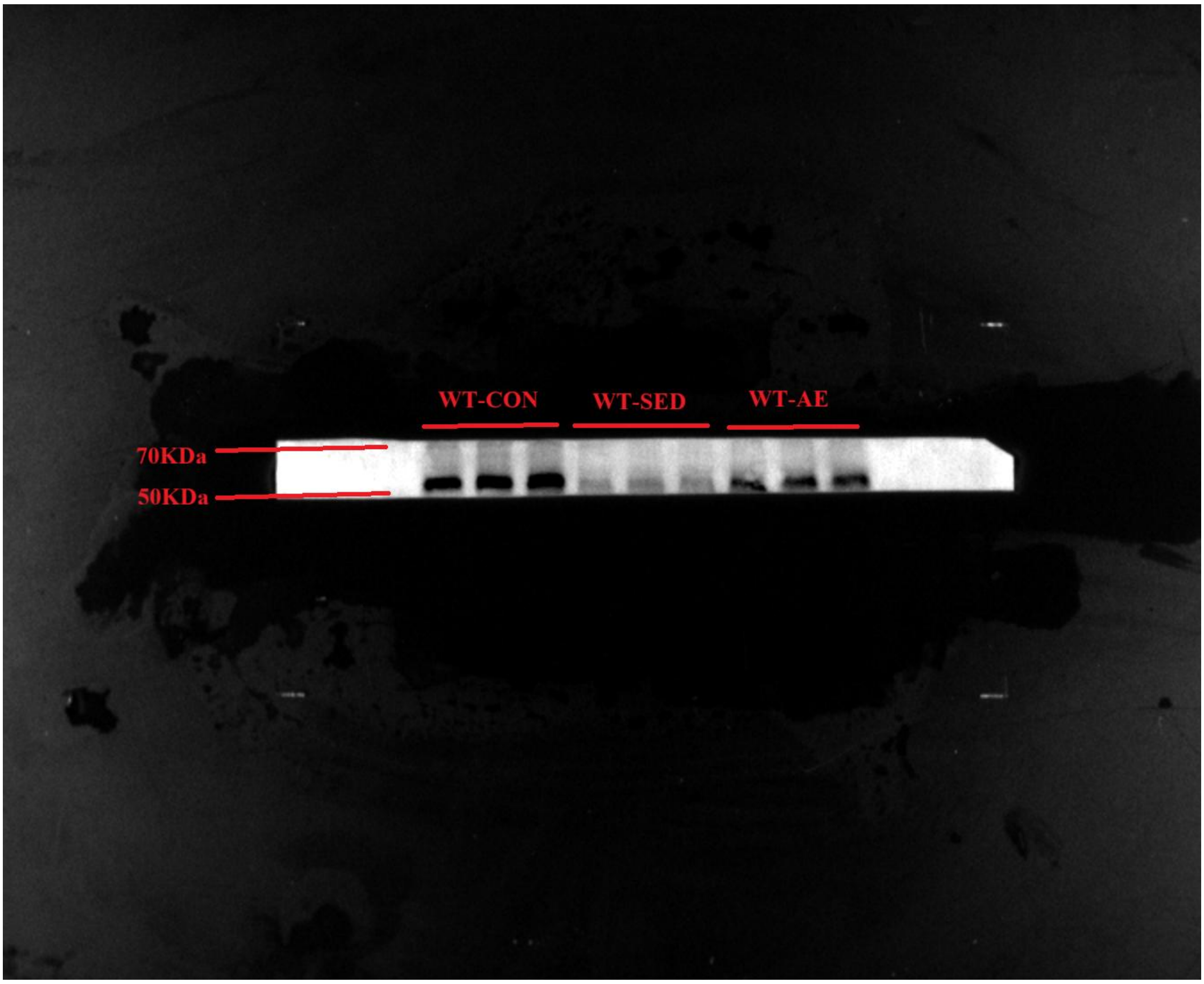

Western blots of β-actin:

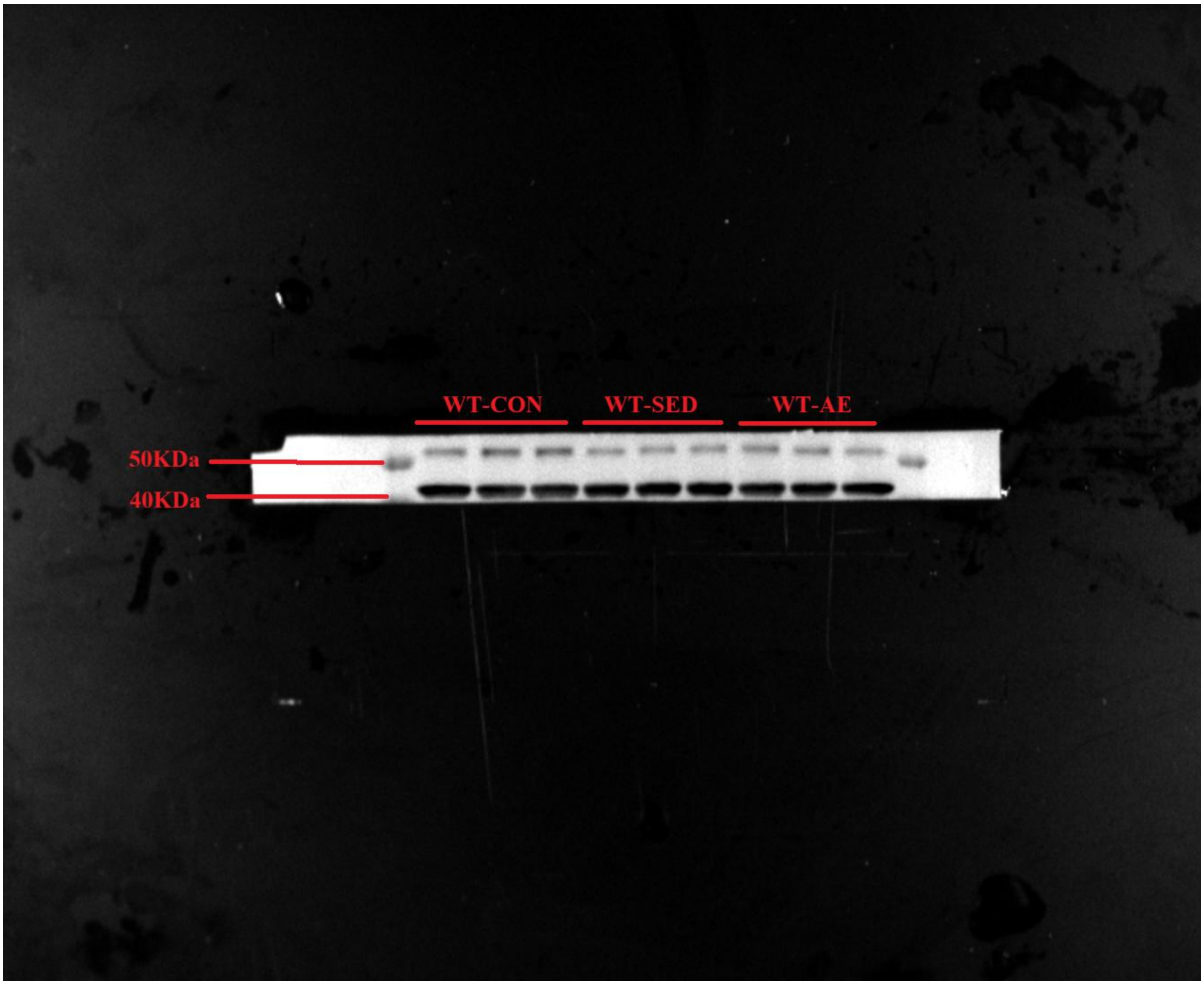

Figure S4P:

Western blots of BDH1:

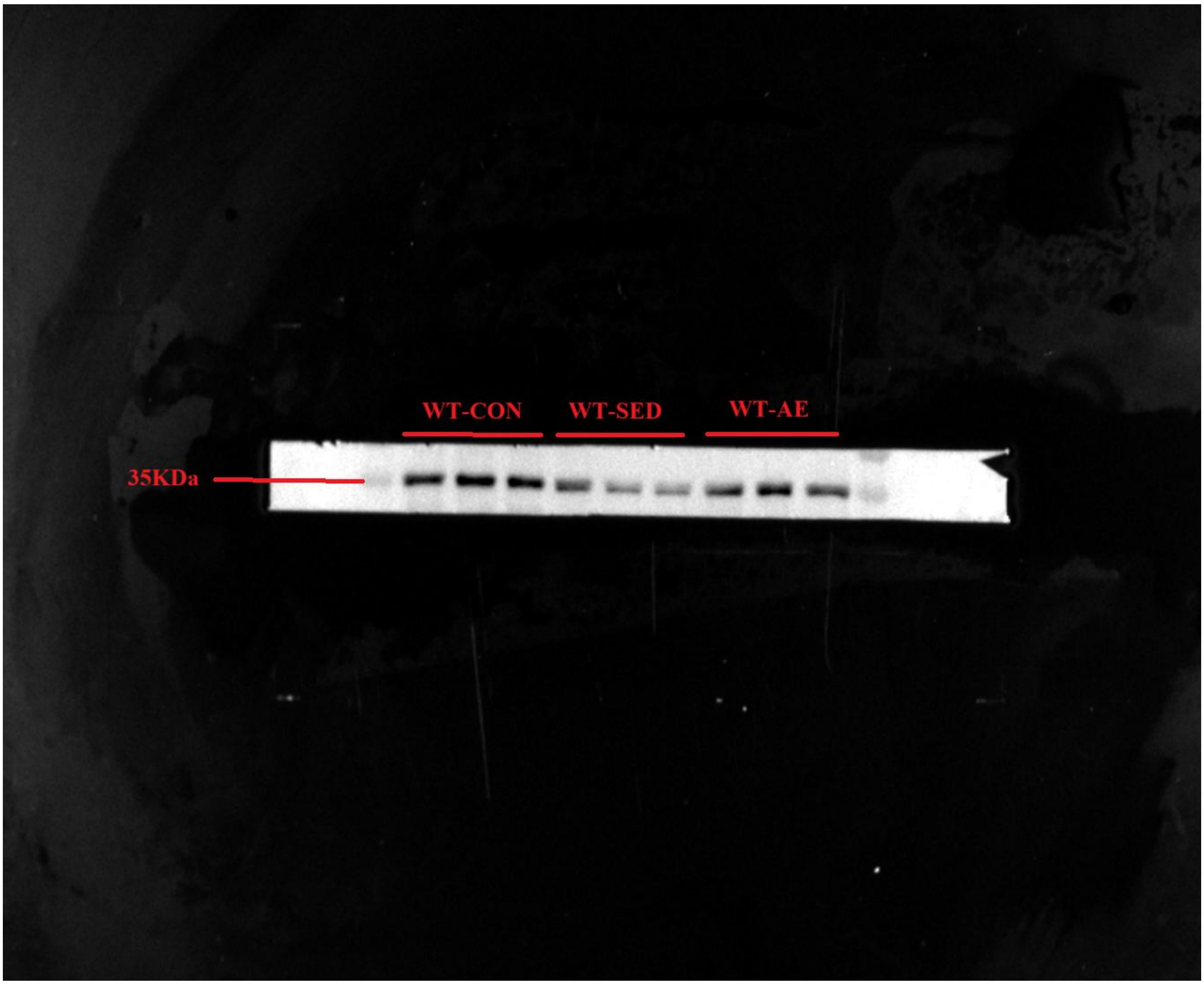

Western blots of β-actin:

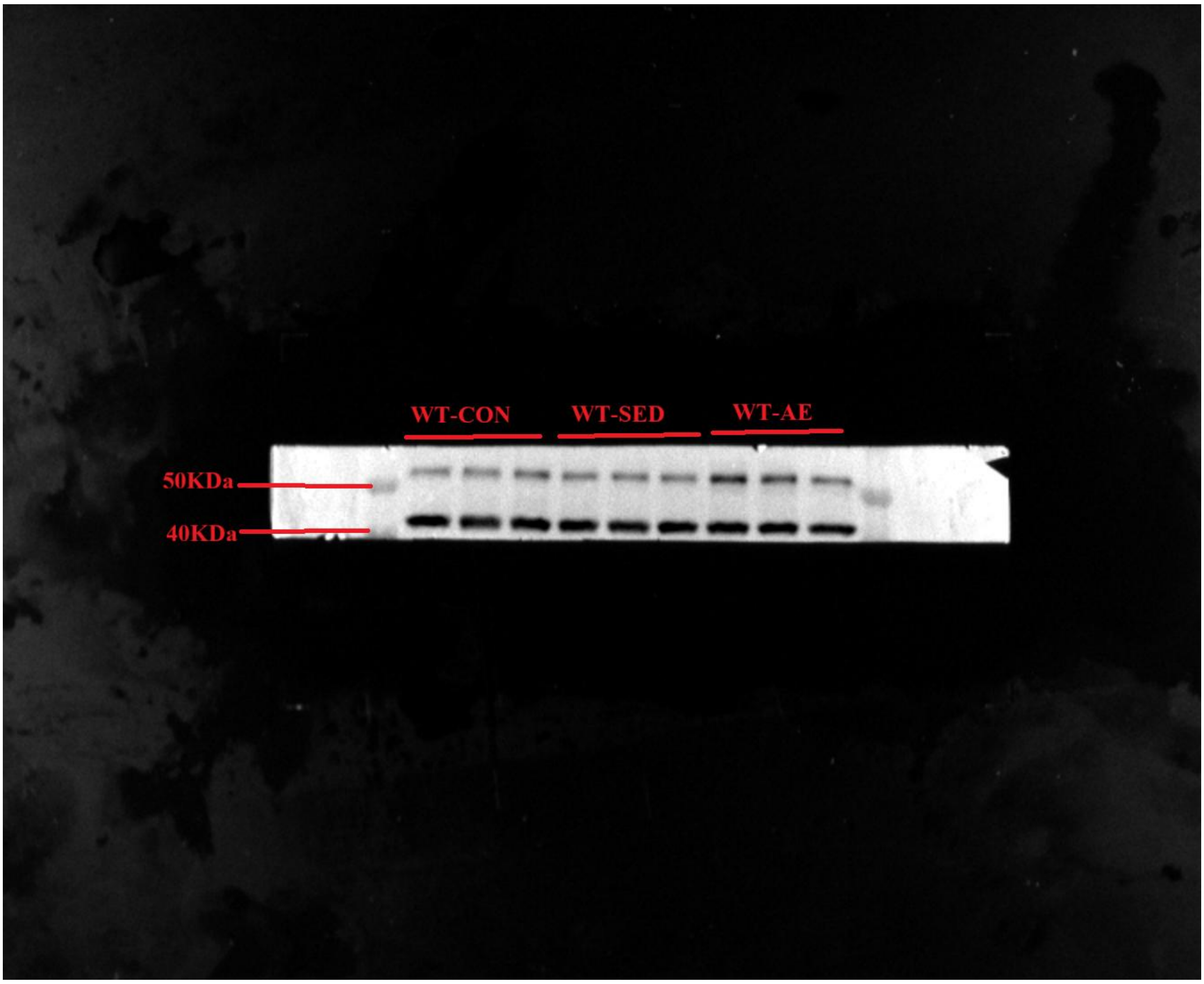

Figure S5B:

Western blots of BDH1:

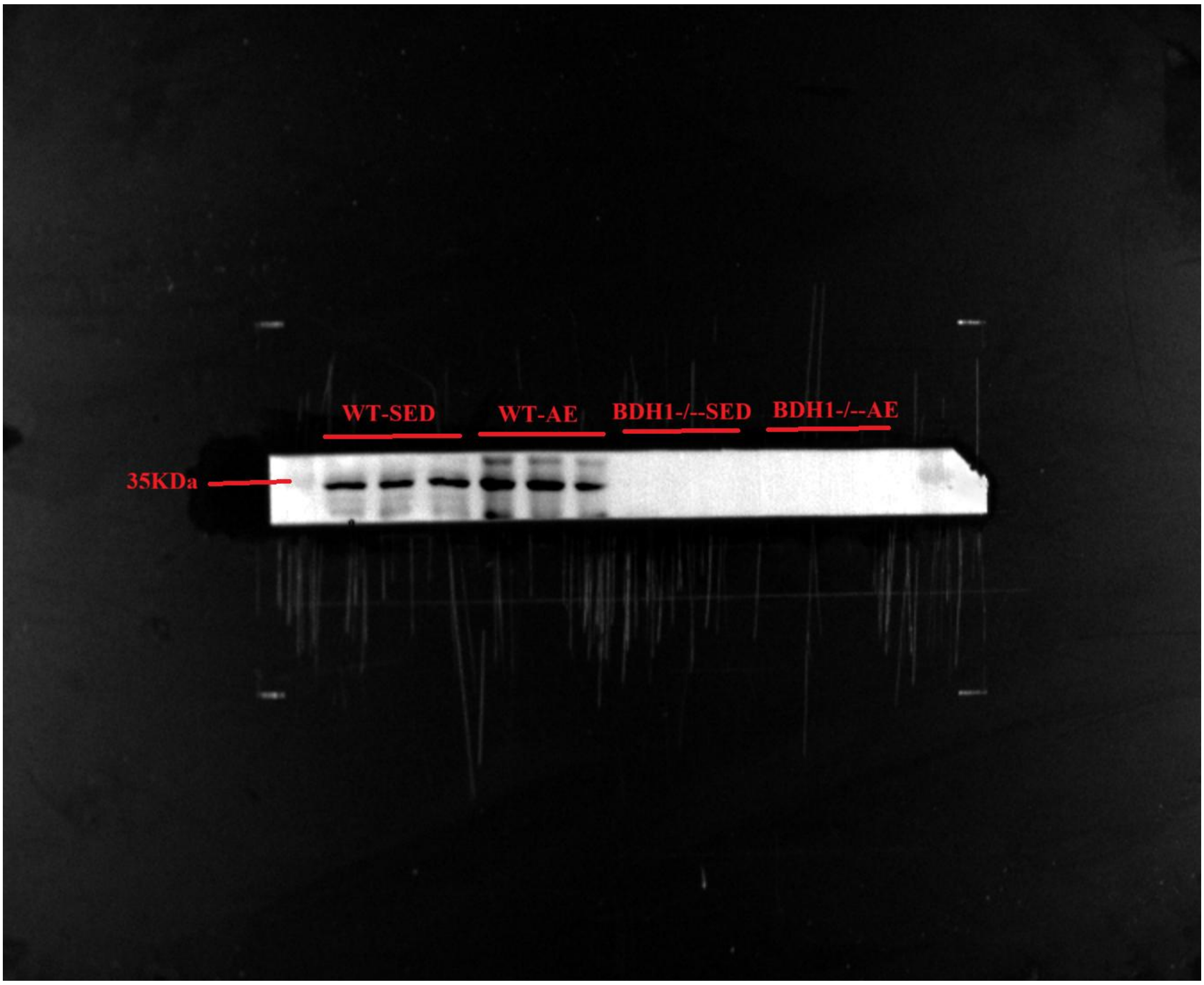

Western blots of β-actin:

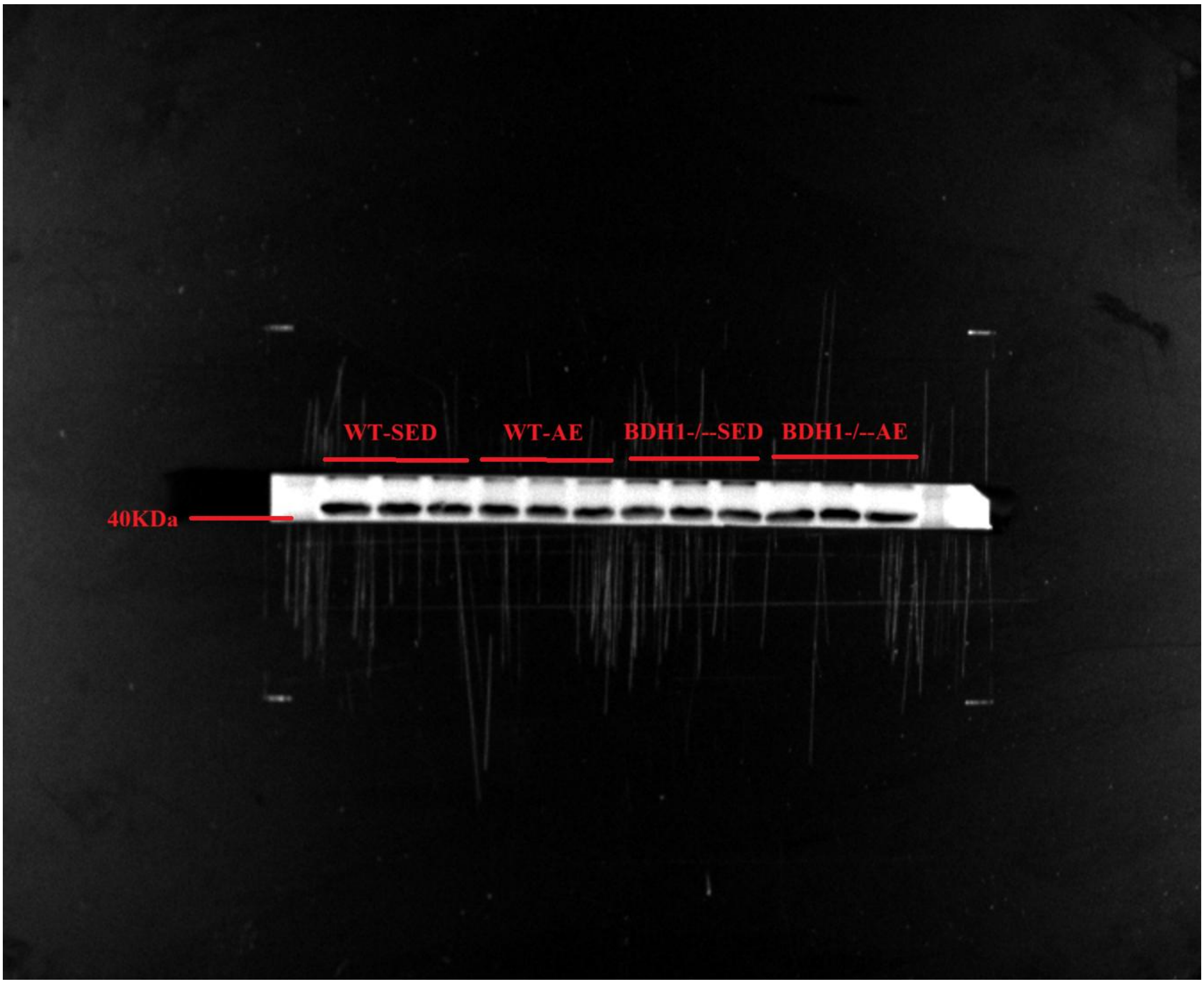

Figure S5K:

Western blots of Nrf1:

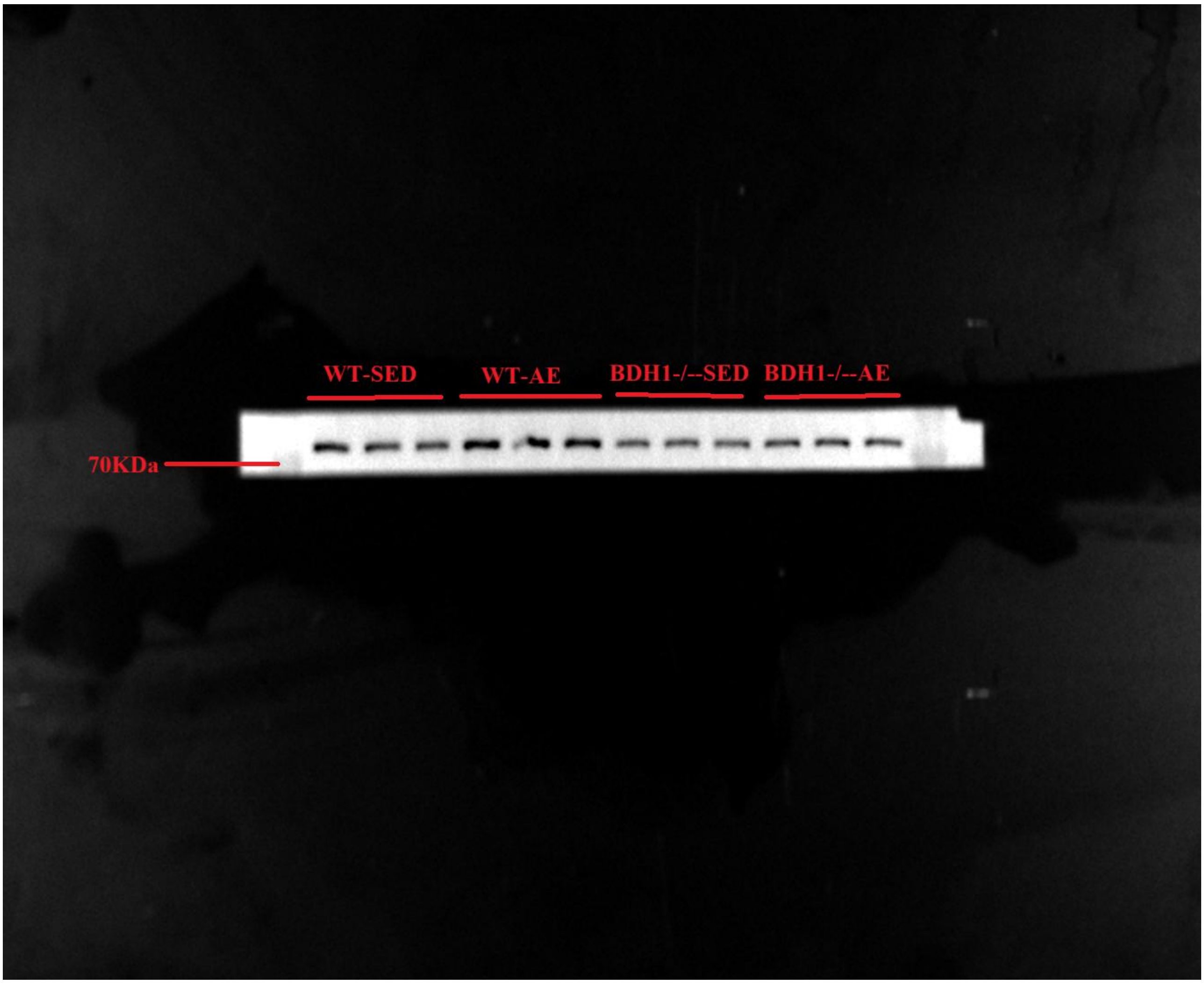

Western blots of Drp1:

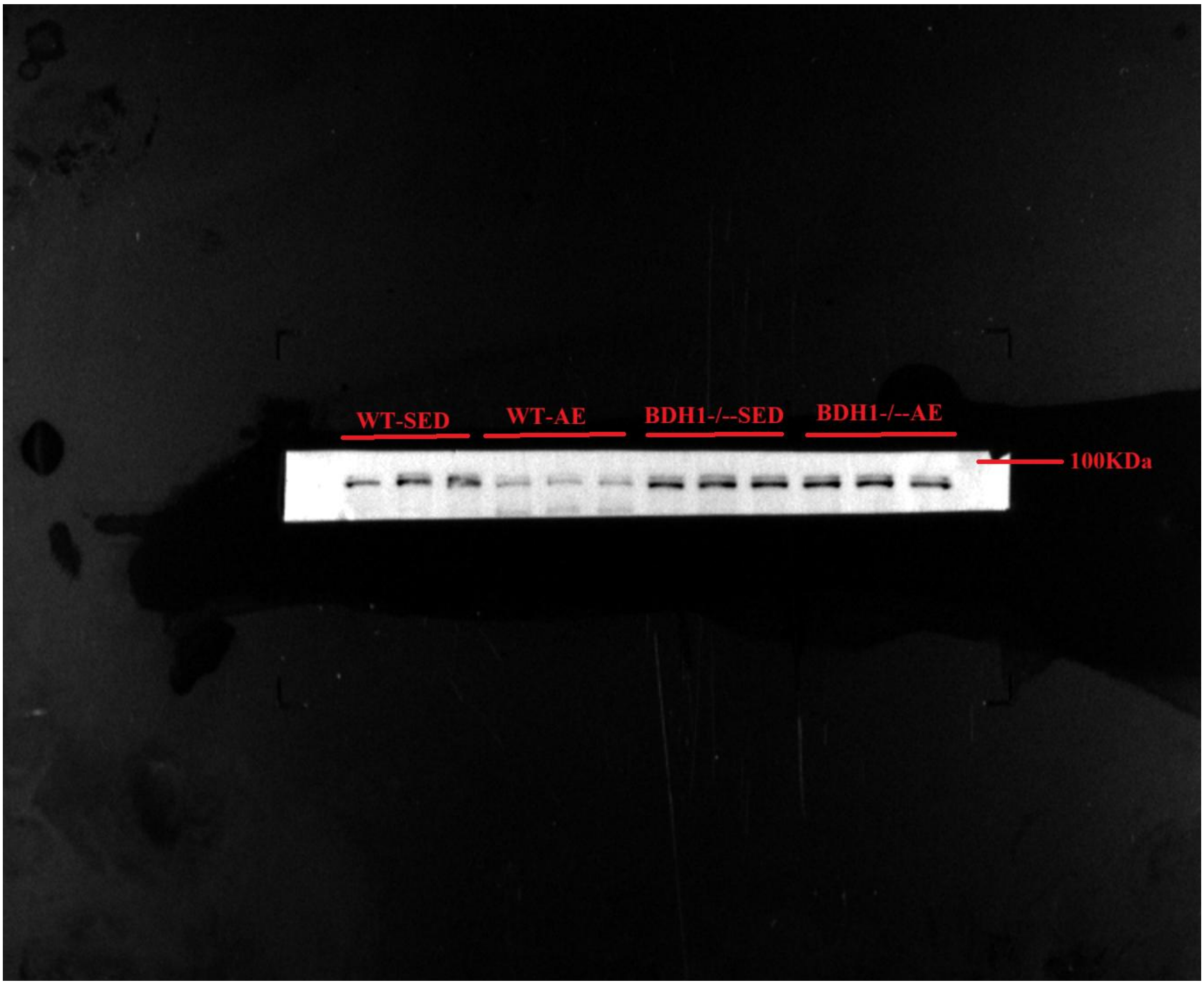

Western blots of MFN2:

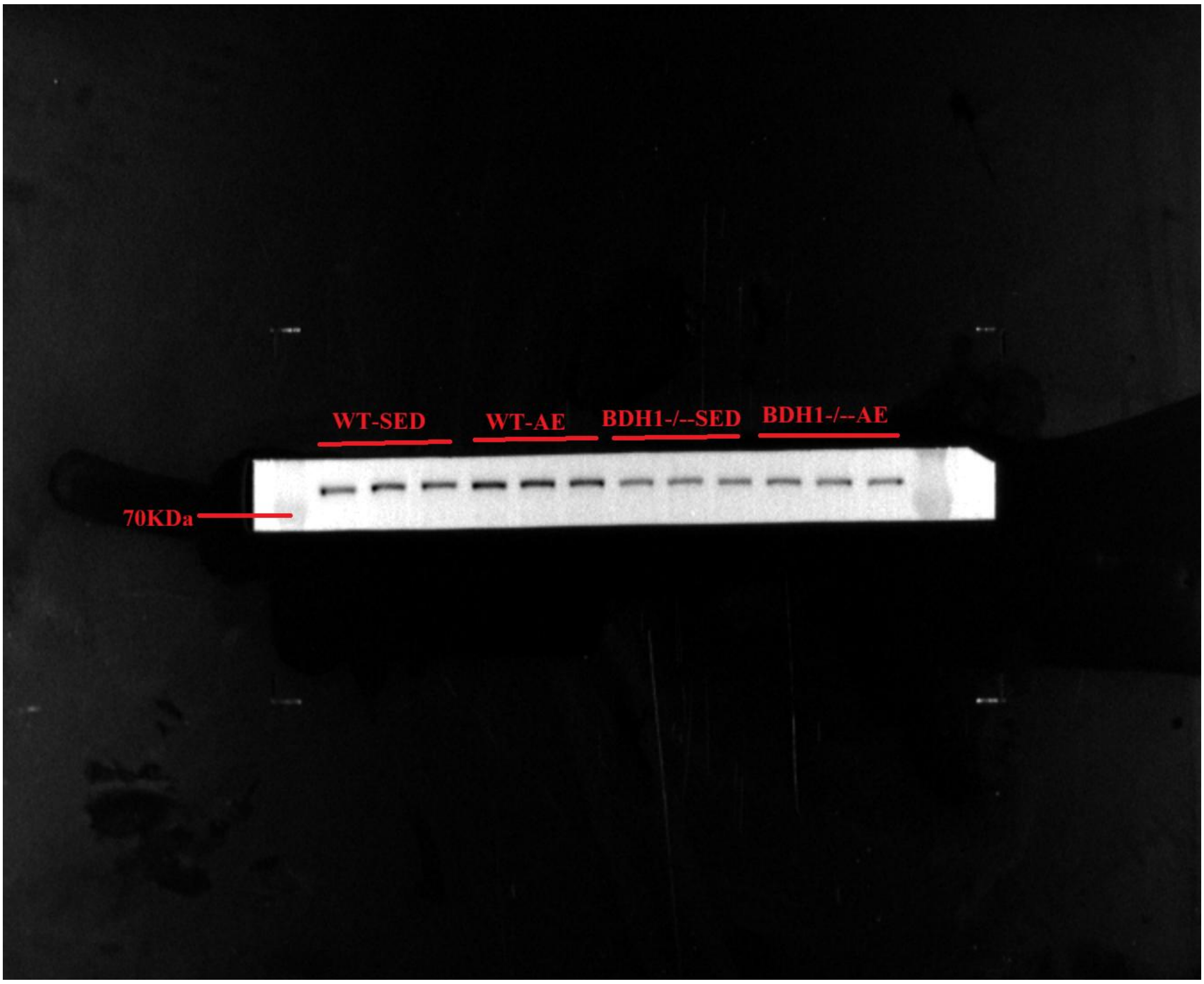

Western blots of OPA1:

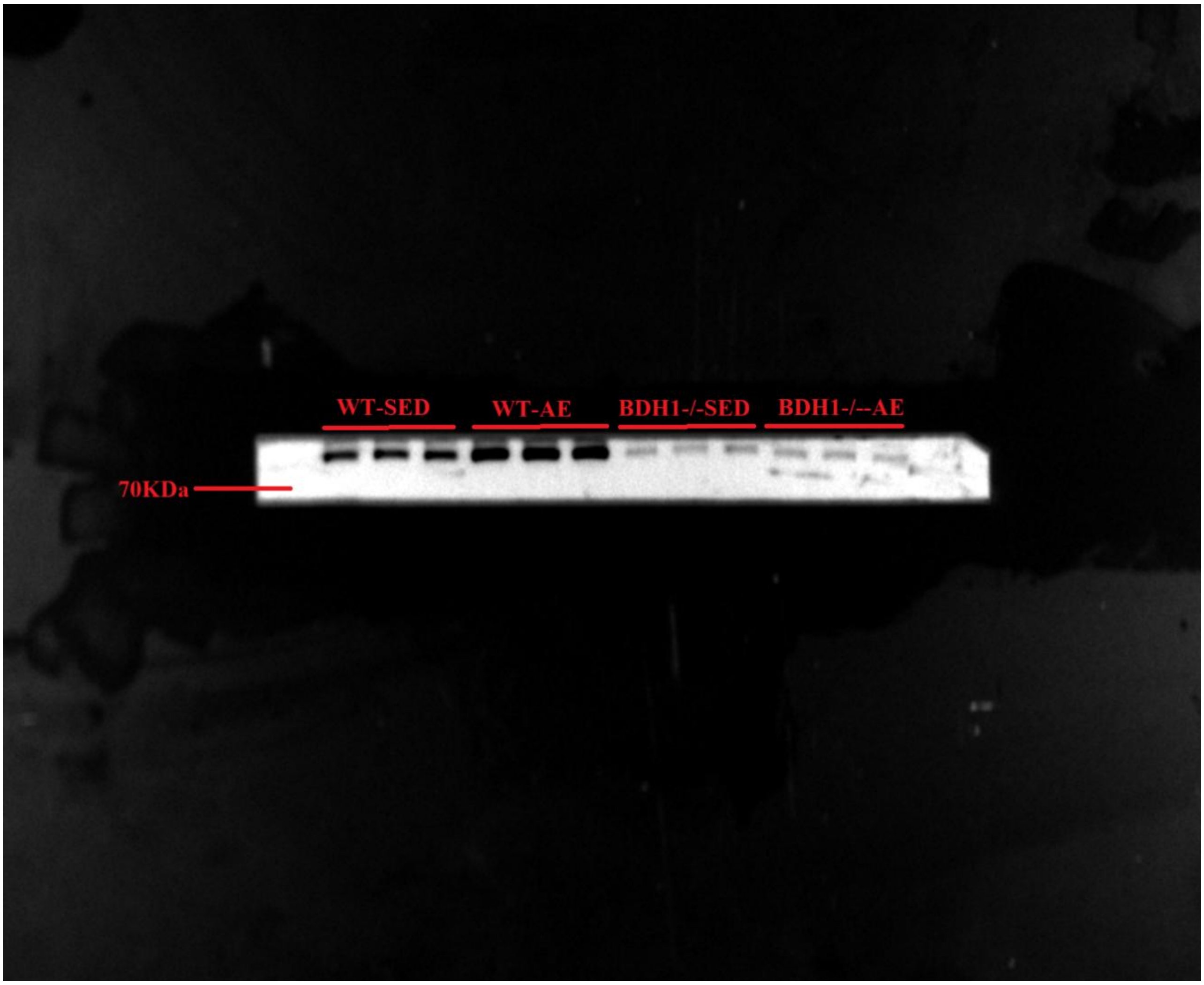

Western blots of β-actin:

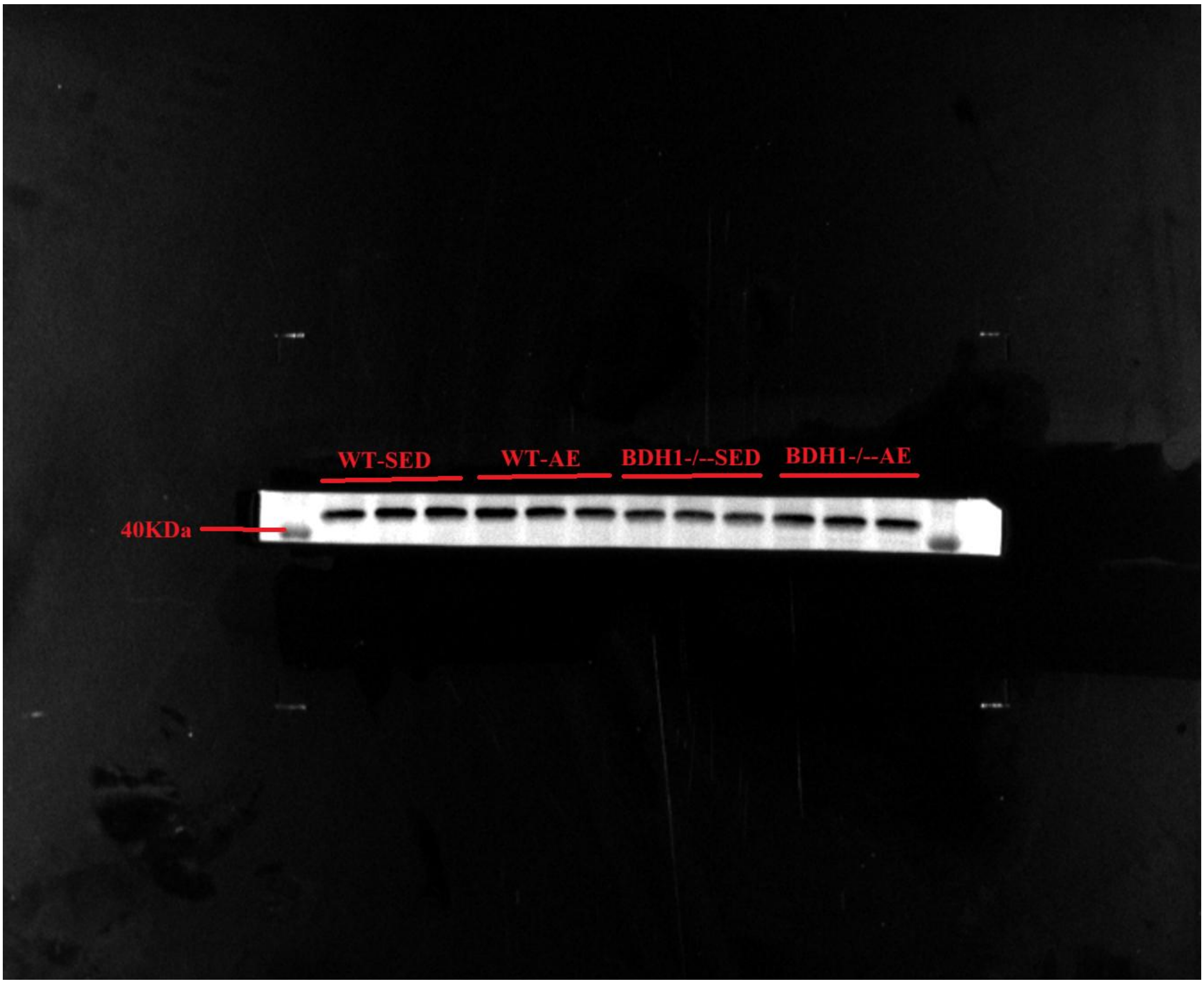

Figure S5S:

Western blots of Nrf2:

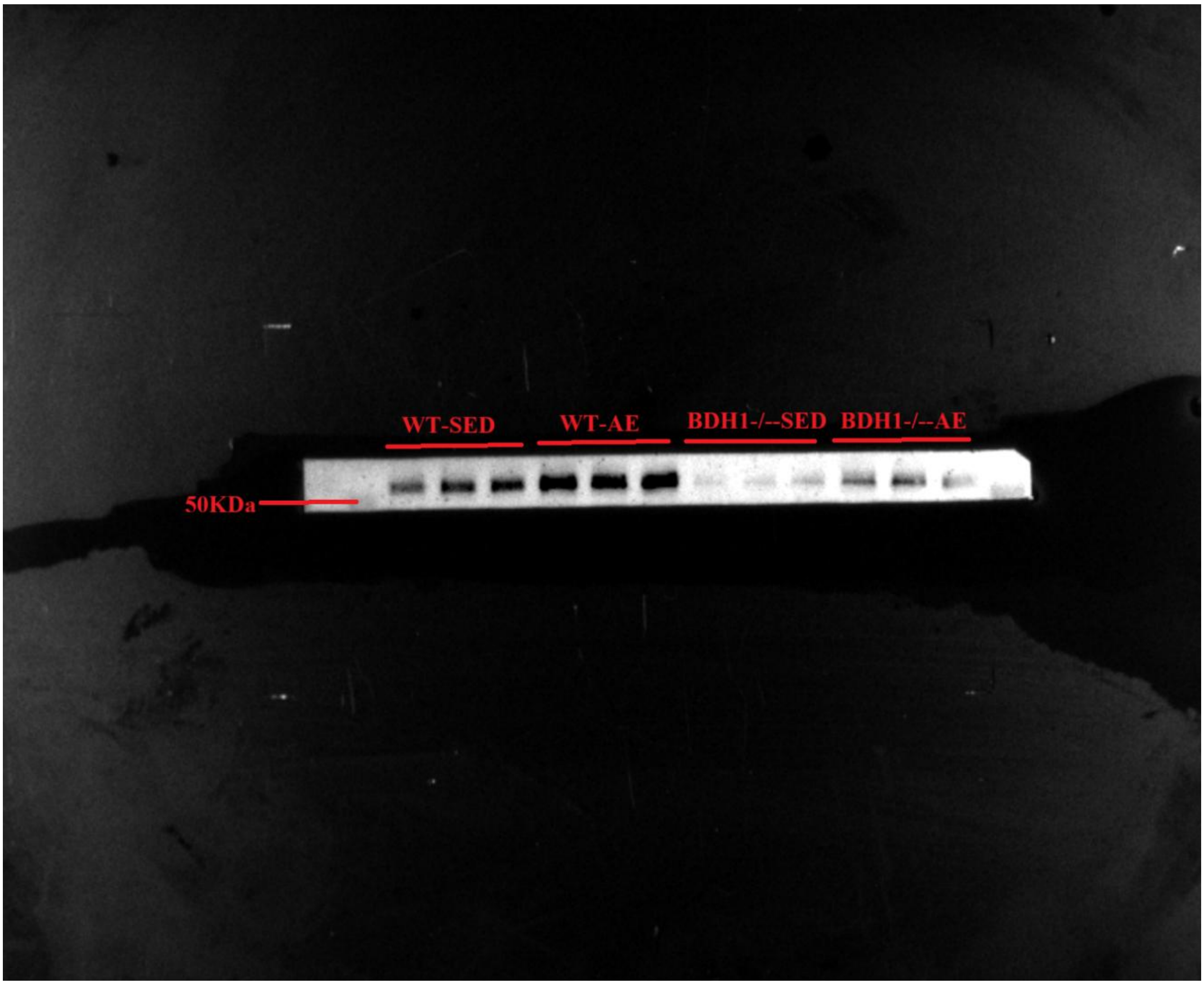

Western blots of β-actin:

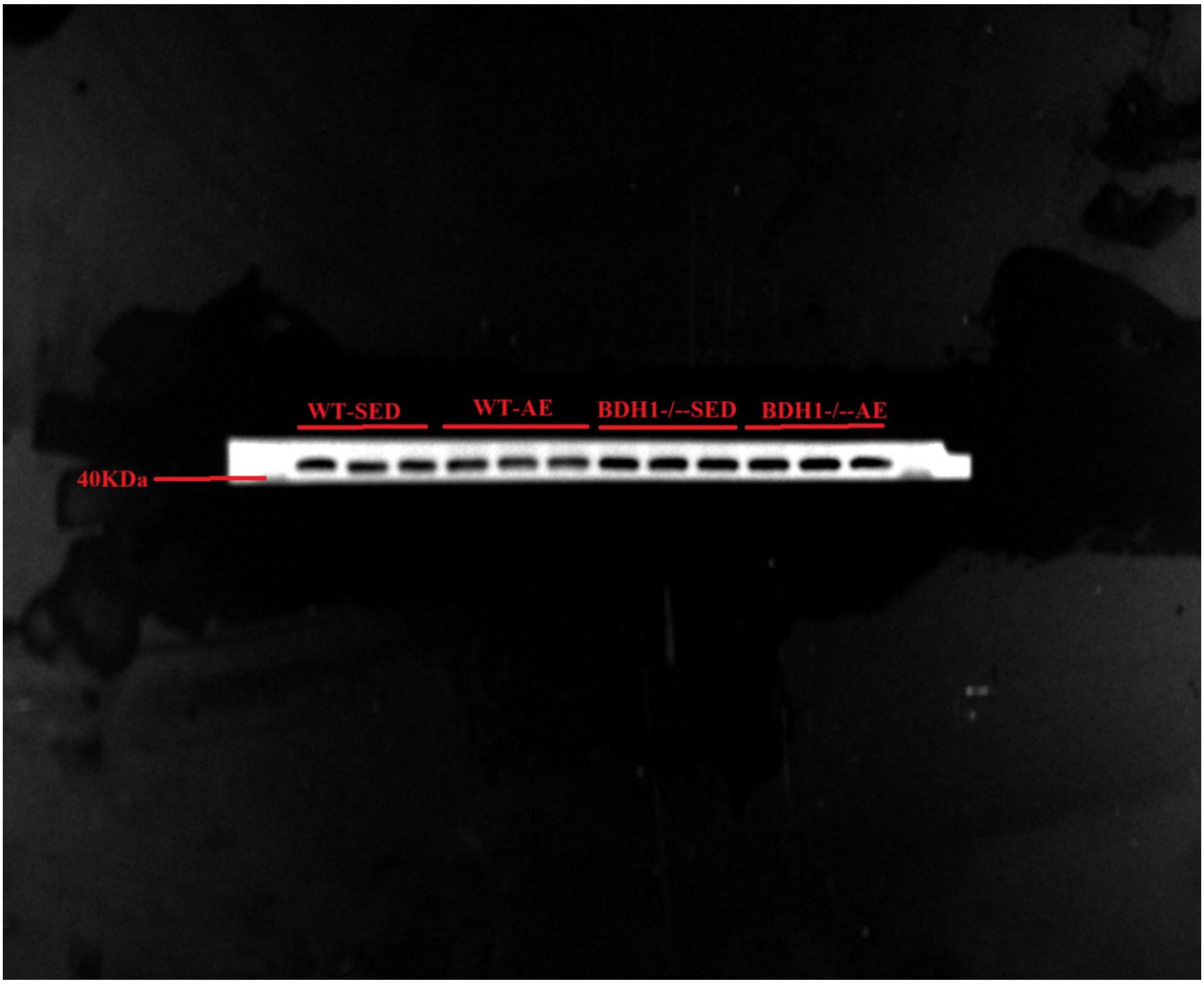

Figure S6I:

Western blots of α-SMA:

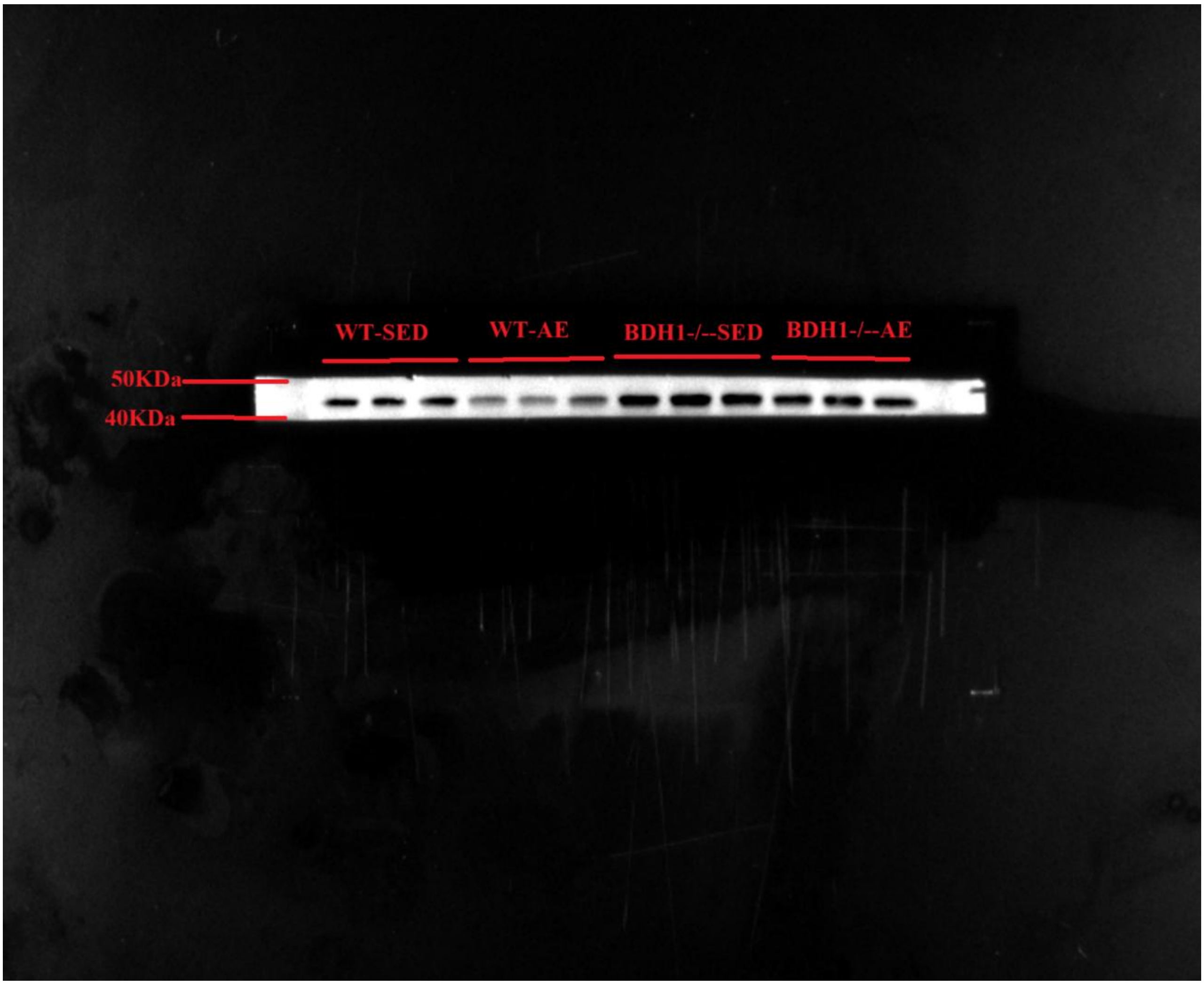

Western blots of β-actin:

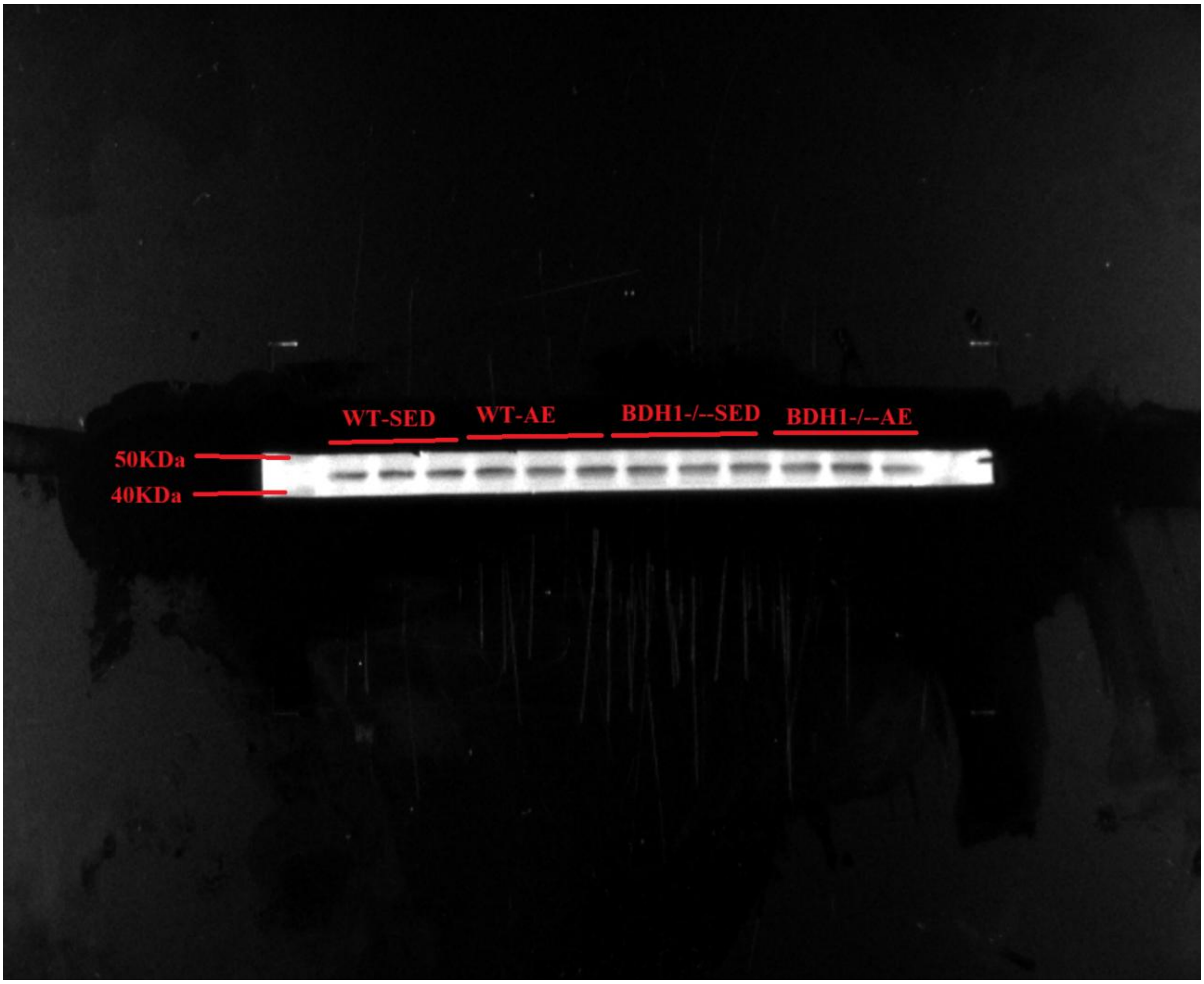

Figure S7E:

Western blots of PI3K:

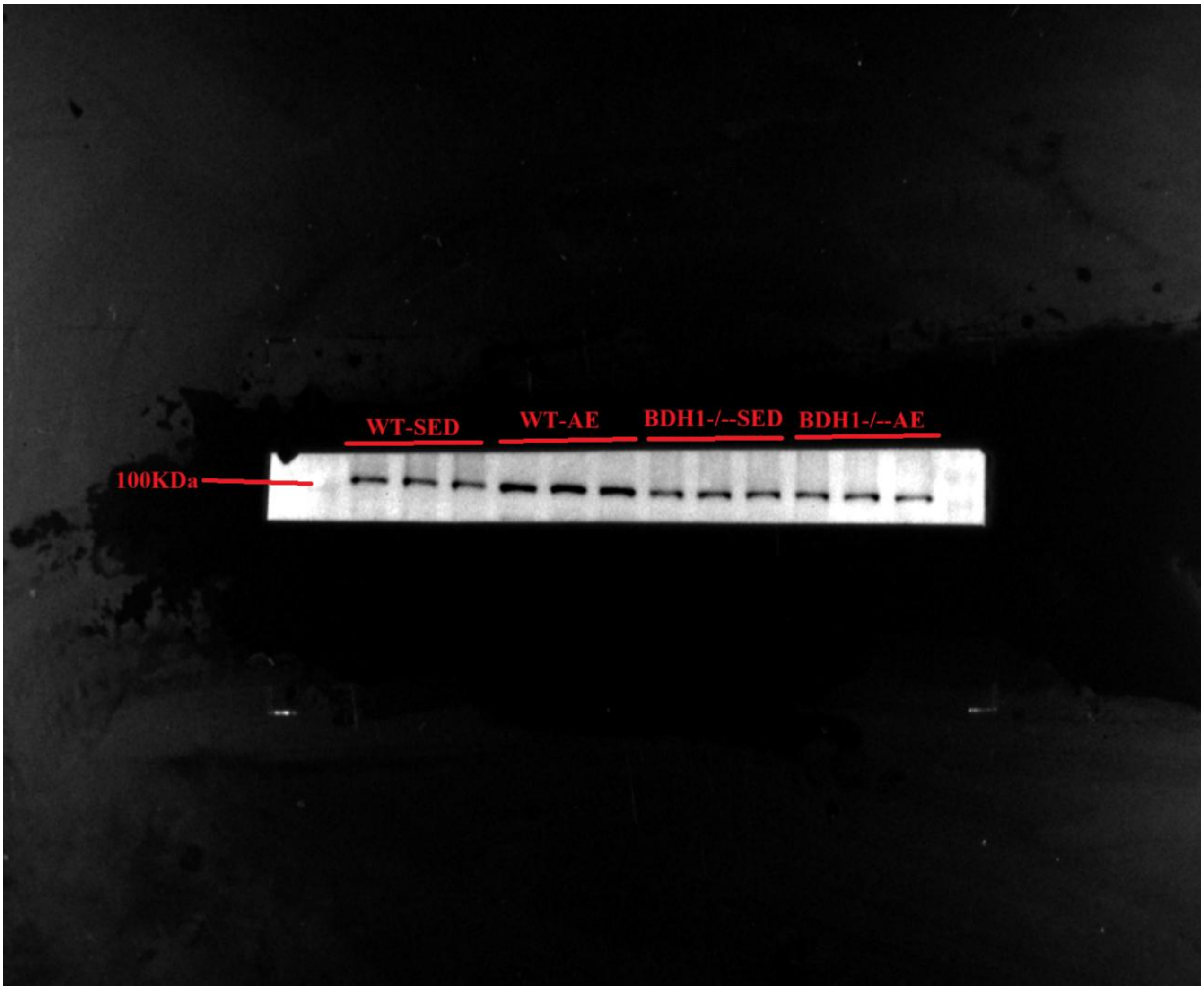

Western blots of Glut4:

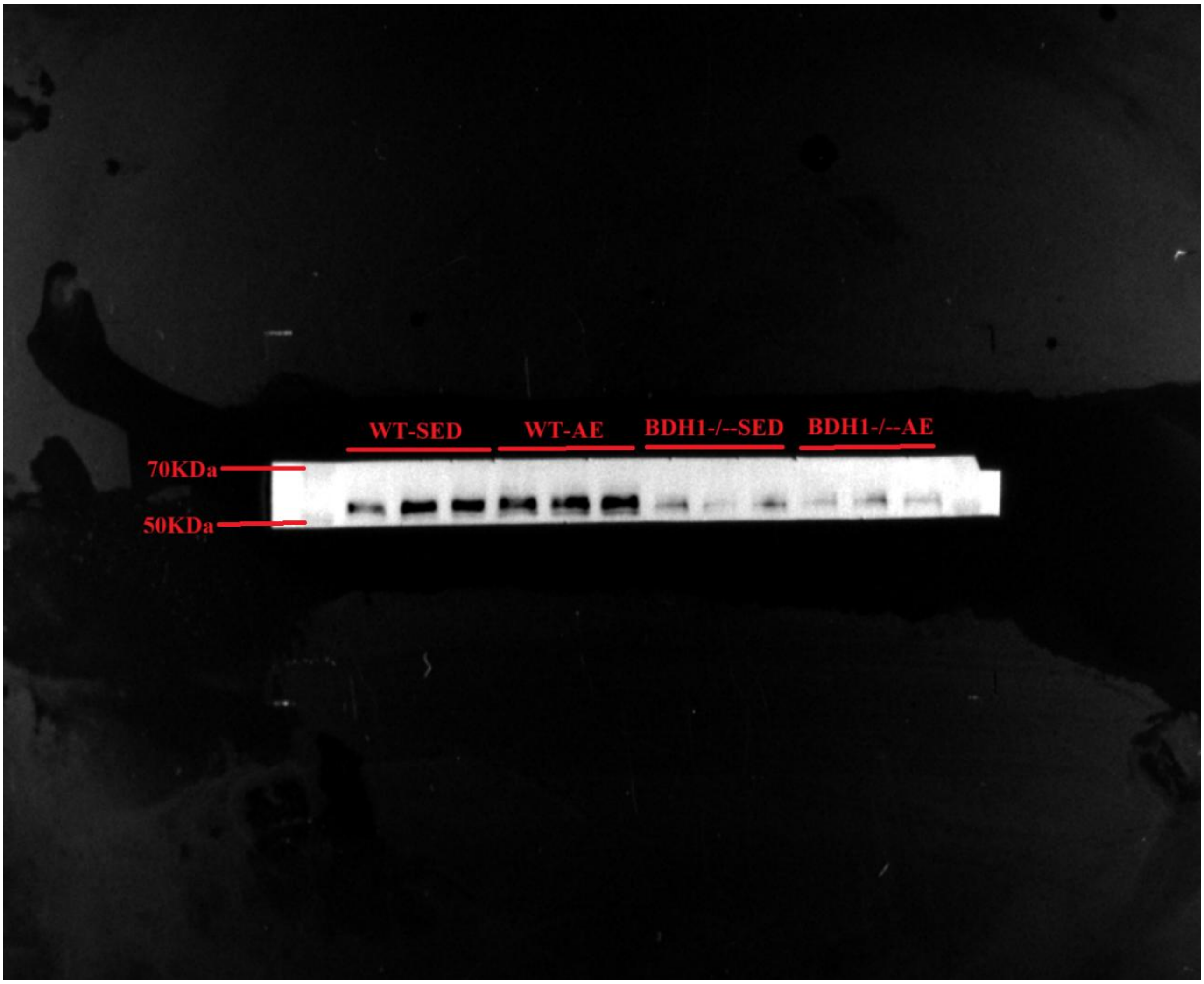

Western blots of β-actin:

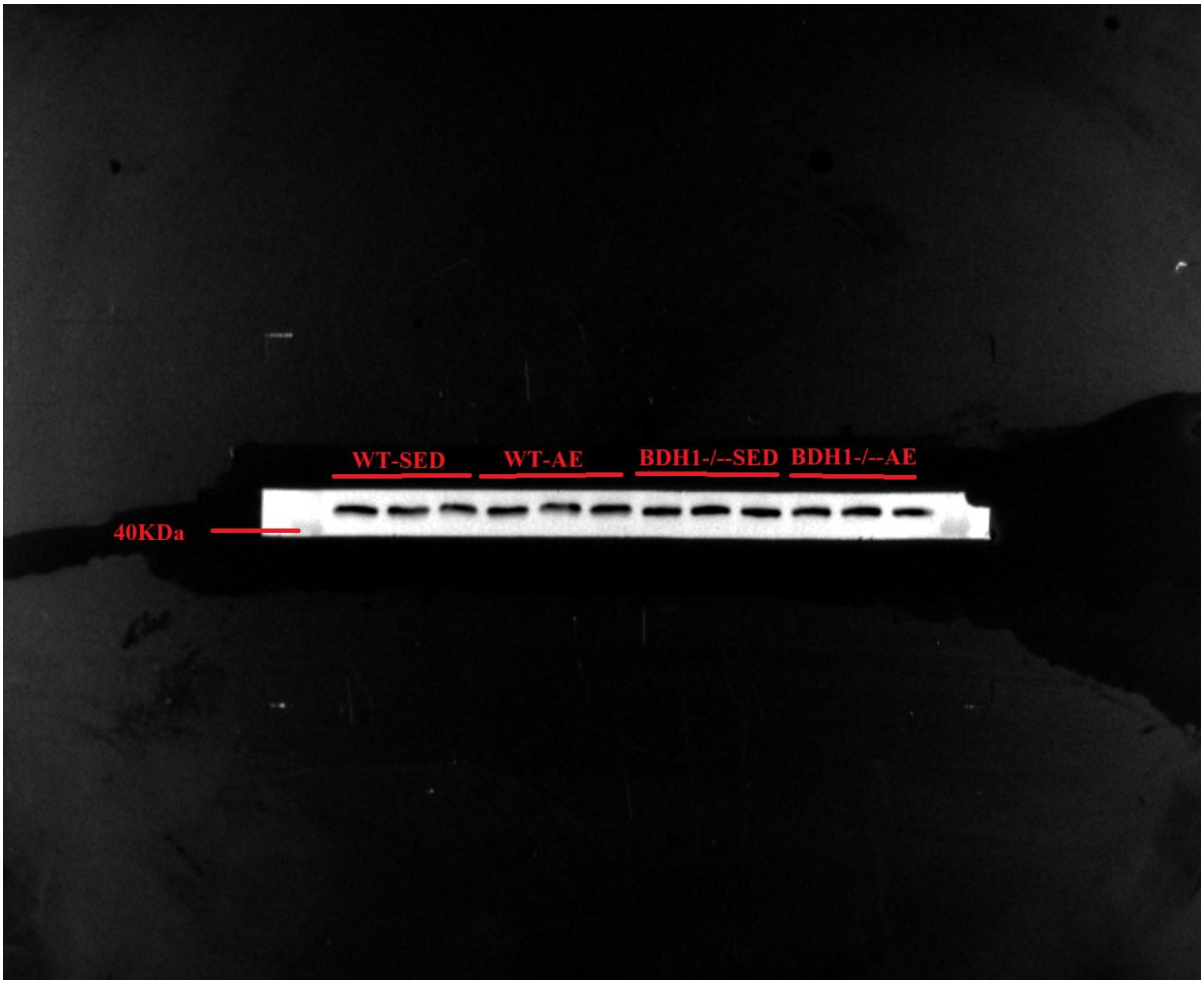

Supplement: Supplementary file 1 [file biomolecules-16-00115-s001.zip › biomolecules-4043221-Supplementary Materials.pdf]
